# Meat analogues: The relationship between mechanical anisotropy, macrostructure, and microstructure

**DOI:** 10.1016/j.crfs.2025.100980

**Published:** 2025-01-21

**Authors:** Miek Schlangen, Iris van der Doef, Atze Jan van der Goot, Mathias P. Clausen, Thomas E. Kodger

**Affiliations:** a Laboratory of Food Process Engineering, Wageningen University, PO Box 17, 6700 AA, Wageningen, the Netherlands; b SDU Biotechnology, Department of Green Technology, University of Southern Denmark, Campusvej 55, DK-5230, Denmark; c Physical Chemistry and Soft Matter, Agrotechnology & Food Sciences Group, Wageningen University & Research, Wageningen, WK 6700, the Netherlands

**Keywords:** Mechanical properties, Meat analogues, Structure, Air bubbles, Anisotropy, Microstructure

## Abstract

Texture of meat analogues is crucial for consumer acceptance, yet it remains poorly defined, but it known that it is influenced by mechanical properties and structure at different length scales. This study describes the relationships between macrostructure, microstructure, and mechanical anisotropy in meat analogues. Two distinct meat analogue product sets are produced with shear cell technology varying in formulations and processing conditions to obtain a wide range of product structures: one based on mung bean protein-rich fractions and the other based on combinations of soy protein isolate and pectin. Mechanical properties are assessed using tensile testing, microstructure is studied using X-ray tomography and confocal laser scanning microscopy, and macrostructure is quantified using a computer vision algorithm based on segmentation and shape features. Both correlation analyses on the response parameters and parameter variance are studied to distinguish the product sets. Strong correlations are found between anisotropy-related parameters, such as fibre score in macrostructure, air anisotropy in microstructure, and the toughness anisotropy index from mechanical properties. Some correlations are found to be product-set independent, such as air bubble anisotropy and fibre score, indicating universal relationships within this study, while other correlations are product-set dependent, such as between fibre score and the anisotropy index of the Young's Modulus in the mung bean fine fraction product set. The relationship between microstructural air bubbles and macrostructure and mechanical properties is apparent in all correlation analyses. Last, univariate feature selection provided insight into which parameters are most important for selected target features.

## Highlights

• Eleven meat analogues divided over two product sets are studied.
• Universal correlations and product-set-specific correlations are found.
• Fibre score, air anisotropy, and toughness anisotropy index are strongly correlated.
• Air bubbles are the main microstructural parameter of importance.
• Mechanical properties alone are insufficient for texture quantification.

## Graphical abstract

**Figure:**
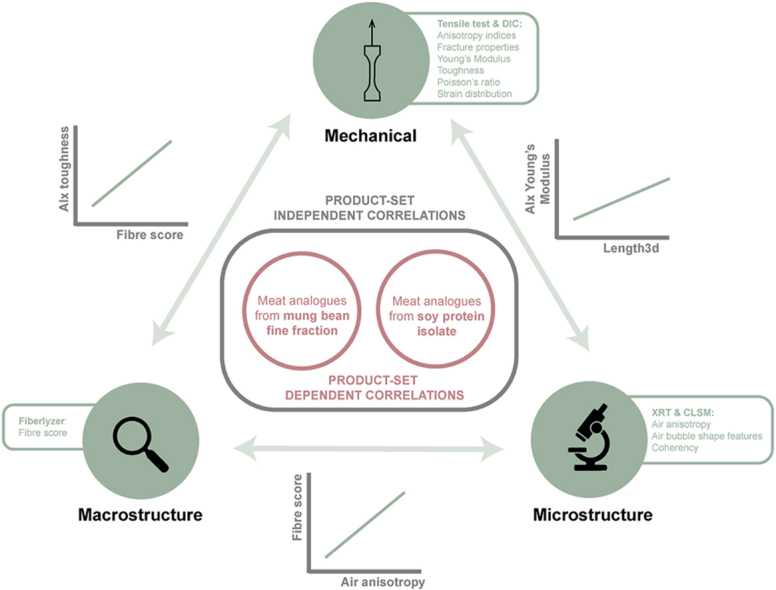

## 1 Introduction

The market acceptance and consumer satisfaction of certain food products, like meat analogues, relies on their texture and structure. Texture has been identified as a key element in consumer acceptability of meat analogues, because consumers want meat analogues that are similar to meat's texture, including its fibrous character (Michel et al., 2021). Comparison of a range of commercially available meat products and their meat analogue counterparts showed that their texture properties (measured mechanically and sensorially) and microstructure are significantly different still (McClements and Grossmann, 2022; Ng et al., 2024; Samard and Ryu, 2019). Understanding texture and structure of these products is therefore essential to improve consumer acceptance and appeal.

Texture is defined as the perception of a food product upon oral processing or consumption; both the mechanical response as well as visual elements are of importance to consumers of food products (Bourne, 2002; Wilkinson et al., 2000). While visual elements create expectations about texture, they may not be considered part of the texture itself. Unfortunately, texture is challenging to quantify, being perceived both tactually and visually. Because texture is poorly defined, a material's quantitative structure, being the arrangement of components in the food product spanning across multiple length scales, is often taken as a starting point for product design. This is justified by the fact that, the structure of meat analogues is strongly correlated with their overall texture (Fu et al., 2023). In the case of meat analogues, structure arises from the thermo-mechanical arrangement of plant proteins with the goal of specifically resembling the structure of animal protein. The structure of animal protein in meat is hierarchical, spanning from macro to micro to nano (Pette and Staron, 1990). However, whether meat analogues exhibit similar hierarchical structures across length scales is unclear. Structuring of plant proteins into meat analogues is rather complex, and significant effort has been dedicated to improving their texture and structure through various different technologies and combinations of ingredients (Ghanghas et al., 2024; McClements, 2024; Snel et al., 2022). Despite the effort, it remains unclear how to effectively measure structure improvement.

The structure of meat and meat analogues has been attempted to be measured extensively, as previously reviewed by McClements et al. (2021) and Schreuders et al. (2021b). Often, these techniques explore only one length scale, infrequently multiple length scales. Mechanical methods include tensile testing and compression testing, both being macroscopic measurements. While stress-strain relationships resulting from these mechanical tests provide information on material properties, they often do not consider the heterogeneity or anisotropy in the products but provide an average over the structural anisotropy. Some studies report on mechanical response of materials in multiple directions, attempting to capture anisotropy, however still averaged over the entire sample (Dahl et al., 2025; Dekkers et al., 2016; Snel et al., 2023). Macrostructure analysis mostly relies on visual qualitative descriptions of the product (Dekkers et al., 2016; Grabowska et al., 2016; Seetapan et al., 2023). Recently, a macroscopic quantitative image analysis method based on computer vision was developed to calculate a fibre score of meat analogues (Ma et al., 2024a, 2024b). On the microscale, anisotropy is qualitatively described based on microscopy images (Cui et al., 2024; Schreuders et al., 2019; Verfaillie et al., 2024; Wittek et al., 2021b; Zhao et al., 2023). A quantitative measure at microscale includes X-Ray Tomography to analyse air bubble volume and shape features of meat analogues (Dekkers et al., 2016; Nieuwland et al., 2023; Snel et al., 2024); not only protein anisotropy, but also air bubble size and shapes likely influence the overall structure (Wang et al., 2019; Zink et al., 2023). Next to air bubbles, the shape and anisotropy of protein aggregates has also been found important in structure analysis. Recently, nanostructure analysis of meat analogues has revealed that the alignment of proteins on nanoscale occurs in the early processing stages of high moisture extrusion cooking (Garina et al., 2024), whereas another study did not find such nanostructure alignment (Guan et al., 2024). Furthermore (Zink et al., 2024), reported that mechanical anisotropy was a direct result of differences in nanometric structure for high moisture extrusion products.

While mechanical anisotropy, determined by for example tensile tests, is a common method for describing meat analogue structures, it does not always correlate with the macrostructural appearance of the products or with the microstructure. In fact (Godschalk-Broers et al., 2022), could not clearly link the mechanical properties of a range of meat analogues to their microstructure as analyzed with confocal laser scanning microscopy. Additionally, the mechanical anisotropy of meat analogues based on pea protein and soy protein did not always correlate with descriptive macrostructure observations (Schreuders et al., 2019). Furthermore, research on the structure of meat analogues typically describes the outcomes of various structural measurements independently, without examining how the structure at one length scale relates to the structure at another length scale, or to the mechanical properties (Zink et al., 2024). described the relation between mechanical anisotropy and structural anisotropy, but only focused on nanostructure. Currently, there are many experimental approaches to measure structural parameters, however, the relation between macroscale and microscale remains unclear. While such multiscale correlation is a universal challenge in materials science, meat analogues present a unique challenge and opportunity to improve customer acceptance as many experimental approaches and data currently exist.

In this work, we aim to establish relationships between measurable quantities of macrostructure, microstructure, and mechanical anisotropy in meat analogues. Products are studied produced from a range of different protein ingredient and biopolymer compositions, and process conditions. We employ a number of widespread experimental approaches and correlate their responses in simplified meat analogues created from protein isolates and protein-rich fine fractions. Therefore, as a first step to gaining mechanistic understanding of the multiscale structures associated with mechanical responses, this paper focusses on establishing parametric correlations between different methods.

## 2 Materials & methods

### 2.1 Materials

Soy protein isolate (SPI) (Supro 500E) was obtained from Solae (Dupont, St. Louis, Mo, USA). SPI was composed of 81.7 wt% (N x 5.7) protein on a dry weight basis measured using a rapid N exceed® analyzer (Elementar, Langensebold, Germany) and had a dry matter content of 91.2 wt%. High-methylated pectin from citrus peel (SLBQ6929V) was obtained from Sigma-Aldrich (Zwijndrecht, the Netherlands) and had a dry matter content of 92.2 wt%. Dehulled mung beans (*Vigna radiata*) were obtained from Vladex (Middelharnis, the Netherlands) and had a dry matter content of ∼91.5 wt%. A mung bean fine fraction was prepared from the dehulled mung beans by milling and subsequent air classification as previously described by (Schlangen et al., 2023a). The obtained mung bean fine fraction (MBFF) had a protein content of 59.2 wt% (N x 5.7) and a dry matter content of 91.8 wt%. Transglutaminase (ACTIVA wm) was obtained from Ajinomoto Co. (Ajinomoto, Tokyo, Japan). The enzyme was composed of 1% transglutaminase and 99% maltodextrin with a reported enzyme activity of 100 U/g. The term transglutaminase (TGase) in this study is used to refer to the enzyme preparation that includes both transglutaminase and maltodextrin.

### 2.2 Preparation of the SPI/pectin products

The first product set consisted of SPI with different concentration of pectin using a constant combined dry matter content of 44 wt% (Table 1). The total weight comprised of 44 wt% biopolymers and 56 wt% demi water. The ratio of SPI and pectin was varied by stepwise replacing the SPI with pectin (0, 1, 2.2, 3.5, and 5 wt%). The blend was prepared by mixing demi water with SPI with a spatula, hydrating the formed dough for 30 min at room temperature, and subsequently mixing the pectin through the dough. The prepared dough was processed into a meat analogue product using the high temperature shear cell (HTSC) (Wageningen University, the Netherlands). Shear-induced structuring with the HTSC was based on a previously developed method by Dekkers et al. (2016). The prepared dough was placed into the pre-heated HTSC at 140 °C and sheared at 39 s^−1^ for 15 min. Additionally, the sample containing 44 wt% SPI and 0 wt% pectin was also processed without shear (0 s^−1^) as a control. Next, the HTSC was cooled down to approximately 50 °C in 5 min, after which the products were taken out and stored in zip loc bags to prevent moisture loss. The products were frozen at −18 °C and thawed before any analysis was performed. Freezing often improves the fibrous structure of the meat analogues (Chantanuson et al., 2022). HTSC products were prepared in triplicate.

**Table 1:** The composition of the high temperature shear cell products. MBFF: mung bean fine fraction. TGase: transglutaminase. SPI: soy protein isolate.

Product code	MBFF (wt.%)	TGase (wt.%)	SPI (wt.%)	Pectin (wt.%)	Water (wt.%)	Temperature (°C)	Shear rate (0 s^−1^)
MBFF-0	39.5	0.5	–	–	60	120	0
MBFF-20	39.5	0.5	–	–	60	120	20
MBFF-39	39.5	0.5	–	–	60	120	39
MBFF-65	39.5	0.5	–	–	60	120	65
MBFF-130	39.5	0.5	–	–	60	120	130
SPI-0-no shear	–	–	44	0	56	140	0
SPI-0	–	–	44	0	56	140	39
SPI-1	–	–	43	1	56	140	39
SPI-2.2	–	–	41.8	2.2	56	140	39
SPI-3.5	–	–	40.5	3.5	56	140	39
SPI-5	–	–	39	5	56	140	39

### 2.3 Preparation of the MBFF products

The second product set consisted of 39.5 wt% MBFF and 0.5 wt% TGase, of which the optimal conditions were found in previous research (Schlangen et al., 2023a, 2023b) (Table 1). TGase was first dissolved in demi water after which the MBFF was mixed through thoroughly with a spatula. Unlike the SPI/pectin blends, no hydration step was used for the MBFF blends. The prepared dough was placed into the pre-heated HTSC at 50 °C for an incubation step of 30 min to allow TGase crosslinking. The incubation parameters were based on optimal conditions from previous research (Schlangen et al., 2023a, 2023b). After incubation, the HTSC was further heated to 120 °C within 5 min. The dough was then shear structured at 120 °C for 15 min at varying shear rates of 0, 20, 39, 65, or 130 s^−1^. Subsequently, the HTSC was cooled down to approximately 50 °C in 5 min, after which the products were taken out and stored in zip loc bags to prevent moisture loss. The products were frozen at −18 °C and thawed before any analysis was performed. HTSC products were prepared in triplicate.

### 2.4 Macrostructural analysis

The macrostructure of the products was quantified using Fiberlyzer: an automated image analysis method for quantitative characterization of visual fibrousness (Ma et al., 2024a). A 4 × 2 cm specimen was cut from the frozen products. Prior to macrostructural analysis the products were thawed and manually bent, resulting in a tear parallel to the shear direction to expose the inner structure. The folded specimen was placed in clamp and images were collected with a digital camera (A6000, Sony, Tokyo, Japan) equipped with a 100 mm F2/8 FE macro lens (Tokina, Tokyo, Japan). The images were manually cropped to a region of interest and the fibre score was generated using the software.

### 2.5 Microstructural analysis (XRT)

Microstructural analysis of air bubbles in the meat analogue products was performed using a GE Phoenix v|tome|x m X-Ray Tomographer (XRT) (General Electric, Wunstorf, Germany). Frozen meat analogue products were cut into specimens of approximately 1 × 2 cm, placed in an Eppendorf tube, and subsequently thawed at room temperature. The tube was placed at 23 mm from the X-ray source and the detector was placed at a distance of 815 mm from the X-ray source, resulting in a spatial resolution of 6 μm. X-rays were produced with a voltage of 75 kV and a current of 80 μA. The produced X-rays passed through the specimen and the intensity was recorded by a GE Dynamic 41|200 detector with 2024 x 2024 pixels resulting in a pixel size of 200 μm. A complete scan consisted of 1500 projections over 360° of which the first and last three images were excluded. One projection consisted of an average of three images over 250 ms. GE reconstruction software (General Electric, Wunstorf, Germany) was used to calculate the 3D structure of the specimen via back projection. The obtained 3D structures were analyzed using the Avizo imaging software (version 2022.2) to obtain multiple microstructural parameters, including the total sample volume, total air volume, volume, shape, and surface area of each air bubble. Additionally, the length, width, breadth, and thickness of each air bubble was recorded along with their orientation in space. The air anisotropy was calculated by dividing the length by the width of each air bubble. A complete description of the microstructural parameters can be found in the Supplementary Materials S1. All air bubbles in a specimen were analyzed and the mean of each parameter was calculated to represent an entire specimen. XRT analysis was performed in duplicate on two separate HTSC products for both MBFF and SPI products.

### 2.6 Microstructural analysis (CLSM)

Confocal Laser Scanning Microscopy (CLSM) was used to study the microstructure of meat analogue products. Frozen meat analogue products (3 mm thick), previously sheared in a circular motion, were cut into specimen of approximately 10 × 15 mm with the 15 mm dimension aligned to the shear flow. These specimen were then cryo-sectioned at −18 °C (Micron CR50-H, ADAMAS-Instruments Corp., Rhenen, The Netherlands) to yield final specimens of 10 × 15 mm and 60 μm-thickness. The cut slices were transferred to a microscope slide, stained by adding 1 μg/ml Rhodamine B solution until the entire slice was covered, and a cover slide was placed on top. The slices were stained at least 1 h prior to CLSM imaging. The slices were analyzed using a Leica Stellaris 5+ DMi8 microscope (Leica Microsystems, Wetzlar, Germany) equipped with a 543 nm HeNe laser and a HC PL APO CS2 10x/0.40 objective. CLSM imaging was performed on three different areas of the HTSC products. The obtained images were analyzed with FIJI and Python as described below to quantify non-protein regions. Quantification involved auto adjusting the brightness and contrast of the images, applying minimum thresholding, and measuring the shape features. Shape analysis was performed on each shape with a size larger than 40 μm and the results were averaged per image. The anisotropy index of the shapes was calculated by dividing the Feret diameter of each shape by the width. To quantify the directionality in the images, the OrientationJ plugin for ImageJ was used similarly as previously described by Clemons et al. (2018). The dominant direction in each image was determined and the coherency to this dominant direction was calculated with OrientationJ. A coherency value of 1 corresponds to a structure that has one dominant direction, which can be interpreted as high anisotropy, while a coherency value of 0 corresponds to a structure without a dominant direction (Gaber et al., 2023).

### 2.7 Mechanical properties

The mechanical properties of the products were analyzed using tensile tests with a texture analyser (TA.XTPlusC, Stable Micro Systems, Surry, United Kingdom). Tensile tests were found most suitable as they provide mechanical insight and are appropriate for the relatively thin HTSC products. The method for tensile testing was based on previous research (Schlangen et al. 2023c). Tensile specimen were cut from the thawed HTSC products with a dog bone-shaped mold with a gauge length of 8.5 mm and a gap width of 20 mm. Three specimens were cut in both the direction parallel and perpendicular to the shearing direction. The width and thickness of the specimens were recorded and considered in the calculations of the tensile parameters. Digital Image Correlation (DIC) was performed during tensile testing to acquire localized strain distributions and Poisson's ratio of the products. Prior to implementing the tensile test, the frontal surface of the specimen was painted with a thin layer of acrylic white paint (Wolkenwit Kleurtester, Flexa, Sassenheim, the Netherlands) and a black speckle pattern was applied on top of this (OK, European Aerosols, Wolvega, the Netherlands). Uniaxial tensile tests were performed at room temperature with a constant deformation rate of 11.4 mm/min until failure. Deformation of the specimen was recorded at 25 fps with a Sony 4K FDR-AX53 video camera equipped with a Zeiss2.0/4.4–88 mm lens. The force and displacement during tensile testing were recorded by the Exponent Connect Software (Stable Micro Systems, Surrey, United Kingdom). The true stress *σ* (Pa) was calculated with equation (1):

(Eq. 1) $$\sigma \;\left(t\right)=\frac{F\left(t\right)}{A\left(t\right)}$$

where *F*(t) is the force and *A*(t) is the cross-sectional area in the gauge section of the specimen. The area *A*(t) is dynamic and is calculated with equation (2):

(Eq. 2) $$A\left(t\right)=\frac{h_{0}}{h\left(t\right)}\times A_{0}$$

where *h*_*0*_ is the initial gauge length at *t* = 0, *h*(t) is the gauge length at time *t*, and *A*_0_ is the initial cross-sectional area of the specimen (width multiplied by thickness). Here, we assume the specimen to be volume conservative, assigning it a Poisson's ratio of 0.5. While this assumption has recently been challenged for anisotropic foods (Schlangen et al. 2023c), quantifying this quantity for each product is prohibitively time consuming, and for comparison purposes a Poisson's ratio of 0.5 remains relevant. An estimation of the effect of different Poisson's ratios can be found in the Supplementary Material (S6).

The true strain ε (−) was calculated with equation (3):

(Eq. 3) $$\varepsilon \left(t\right)=\ln\frac{h\left(t\right)}{h_{0}}$$

The fracture point was defined as the point where the stress reached a maximum. The Young's Modulus was defined as the slope of the linear part of the stress-strain curve. The fracture length was calculated with equation (4):

(Eq. 4) $$fracture\;length=\frac{\varepsilon_{failure}}{\varepsilon_{fracture}}$$

where ε_failure_ is the strain at the failure point when the stress has reached 0 and ε_fracture_ is the strain at the fracture point. The toughness was defined as the area under the stress-strain curve and it describes how much energy the product can absorb until failure. The toughness was calculated with equation (5):

(Eq. 5) $$Toughness=\int_{0}^{\varepsilon_{failure}}\sigma ,d\varepsilon$$

where ε_failure_ is the strain at the failure point when the stress has reached 0, *σ* is the true stress, and ε is the true strain.

### 2.8 Quantification of parameters from DIC analysis

DIC analysis was performed with the Ncorr2 and Ncorr_Post software as previously described by Schlangen et al. (2023c). The dynamic Poisson's ratio from DIC, *v* (t), was calculated with equation (6):

(Eq. 6) $$v\;\left(t\right)=\frac{\Delta \varepsilon_{transverse}}{{\Delta \varepsilon }_{axial}}$$

where ε_transverse_ is the strain in the transverse direction and ε_axial_ is the strain in the axial direction. The decrease in Poisson's ratio was calculated by equation (7):

(Eq. 7) $$Decrease\;in\;Poisson^{\prime }s\;ratio=v_{t=linear}-v_{t=failure}$$

where *v*_*t = linear*_ is the first Poisson's ratio recorded (excluding scatter outliers) and *v*_*t = failure*_ is the last Poisson's ratio recorded (just before failure).

Furthermore, the strain distribution of the specimen right before fracture was quantified using a spatial autocorrelation method called Global Moran's I. This method can be used to quantify spatial heterogeneity and has previously been used to quantify spatial heterogeneity of elastic modulus of meat analogue samples (Boots et al., 2021). Global Moran's I is defined as:

(Eq. 8) $$I=\frac{N}{W}\frac{\sum_{i}\sum_{j}w_{ij}\left(x_{i}-\bar{x}\right)\left(x_{j}-\bar{x}\right)}{\sum_{i}{\left(x_{i}-\bar{x}\right)}^{2}}$$

where *I* is the Global Moran's I, *N* is the number of cells, *x* is the value of strain as measured by DIC of the cell of interest, $\bar{x}$ is the spatial mean of *x*, *w*_*ij*_ is a spatial weights matrix and *W* is the sum of all *w*_*ij*_. As the strain distribution in this study varied mostly along the axial tensile direction, a horizontal weights matrix was found to align the best with the results, which was equivalent to:

$$w_{ij}=\left(\begin{array}{ccccc} 0 & 0 & 0 & 0 & 0\\ 0 & 0 & 0 & 0 & 0\\ 1 & 1 & 0 & 1 & 1\\ 0 & 0 & 0 & 0 & 0\\ 0 & 0 & 0 & 0 & 0 \end{array}\right)$$

Global Moran's I decreases as the neighborhood distance increases. This decrease corresponds to a decrease in local correlation in strain and thus defines a length scale for strain heterogeneity. The maximum range used in the Global Moran's I analysis was 8. A distance of 1 was equal to 37 μm in real space. The Global Moran's I was plotted against the neighborhood distance and a linear fit was applied to the data. The slope of this fit was recorded and used as parameter to describe heterogeneity of the strain distribution.

As DIC analysis is relatively high in computational intensity, only one representative specimen per formulation was processed. The representative specimen was chosen based on expert judgement of the recorded videos, with specimen preferably chosen where the fracture was on the side of the specimen oriented towards the camera.

### 2.9 Correlation analysis, variance analysis, univariate feature selection, and data availability

Pearson's correlation tests, variance analysis, and univariate feature selection were conducted using the NumPy, Pandas, Matplotlib, SciKit, and Seaborn libraries in the Python programming language. Prior to parameter variance analysis and univariate feature selection, normalization of the data was performed to ensure consistency in scale using the MinMaxScaler() from the sklearn package. Univariate feature selection was performed with the SciKit library using f_regression and selecting the top 3 (k = 3) features. Considering the size and quantity of the data, we have chosen to publish all data open source in a data repository.

## 3 Results & discussion

### 3.1 Product-set independent correlations

**Fig. 1:**
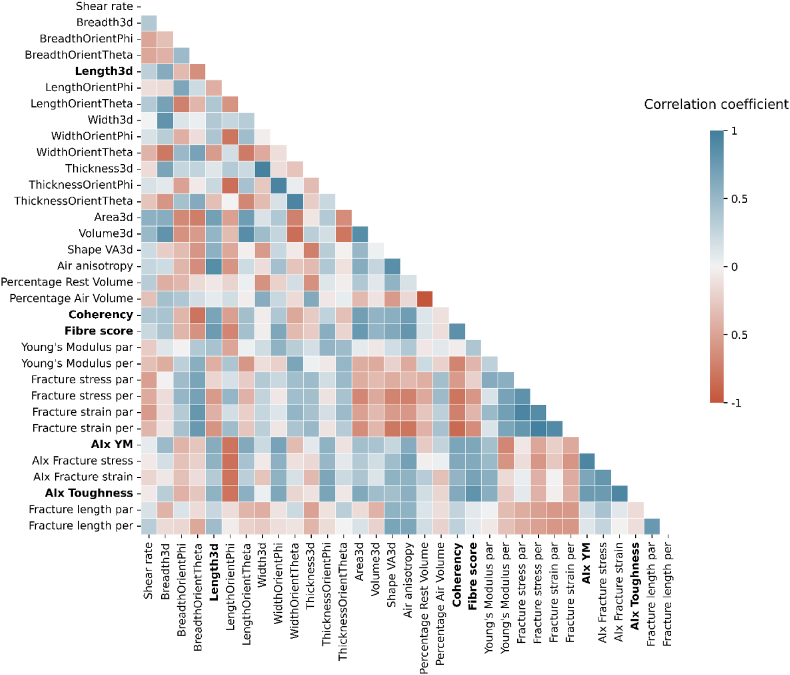
Correlation matrix of entire data set based on averages. Parameters in bold are further highlighted in Fig. 2. AIx refers to anisotropy index, par refers to measured in the parallel direction to the shear flow, and per refers to measured in the perpendicular direction to the shear flow.

Two different meat analogue product sets were prepared tailored to ensure a range of different products (Table 1). These sets were produced using different ingredient combinations and processing conditions to determine if structure is inherently linked to a specific product set or if it is a more general characteristic. Furthermore, the conditions used ensured different products within and among the product sets, as judged visually and tactually by experts and based on preliminary experiments. Meat analogue products comprised of mung bean fine fraction (MBFF) and soy protein isolate (SPI) are measured with many experimental approaches and their responses correlated. The correlation analyses are conducted on both the combined product sets (MBFF and SPI) and on each product set separately. Importantly, a “product set” in this work is defined as meat analogue samples containing either MBFF or SPI. Therefore, product-set independent correlations refer to those identified across both combined product sets, while product-set dependent correlations refer to those found by performing the correlation analysis on just one of the product sets. A description of all response parameters used in this study can be found in the supplementary materials. Although some parameters are intrinsically correlated and others are not, our aim in this work is to demonstrate the process without bias. Therefore, we correlate all parameters. Correlation analysis of the mechanical and structural parameters provides evidence of Pearson correlations, showing correlations based on averages (Fig. 1). An alternative analysis that considers some sample variation is found in the supplementary materials (Fig. S2). The strongest correlations are found between parameters measured using the same technique, for example fracture strain and fracture stress measured using tensile testing (Fig. 1). These correlations are intuitive, because fracture stress and strain are closely interconnected.

More insightful are correlations between differing measuring techniques, as those may measure different properties of the products. Correlations are found across different length scales (between macroscale and microscale) and between mechanical and structural properties (Fig. 1). The latter highlights that structure plays an important role in mechanical performance of a product. The parameters with strong correlations in Fig. 1 are important in texture analysis of meat analogues, irrespective of product set variables such as process conditions or ingredient concentrations. These stronger correlations likely show fundamental relationships governing the behaviour of meat analogues.

Some of the parameters show clustered correlations, such as for air anisotropy and mechanical properties (Fig. 1). Air anisotropy demonstrates positive correlations with all anisotropy indices of mechanical properties, but negative correlations with fracture strain and fracture stress. This indicates that higher air anisotropy corresponds to lower mechanical parameters. This finding supports previous research on meat analogues produced with the shear cell, which also found positive correlations between mechanical anisotropy at fracture and air bubble deformation (Schreuders et al., 2019; Snel et al., 2024; Wang et al., 2019), and suggest that elongated air bubbles likely increase the probability of the sample to fracture.

**Fig. 2:**
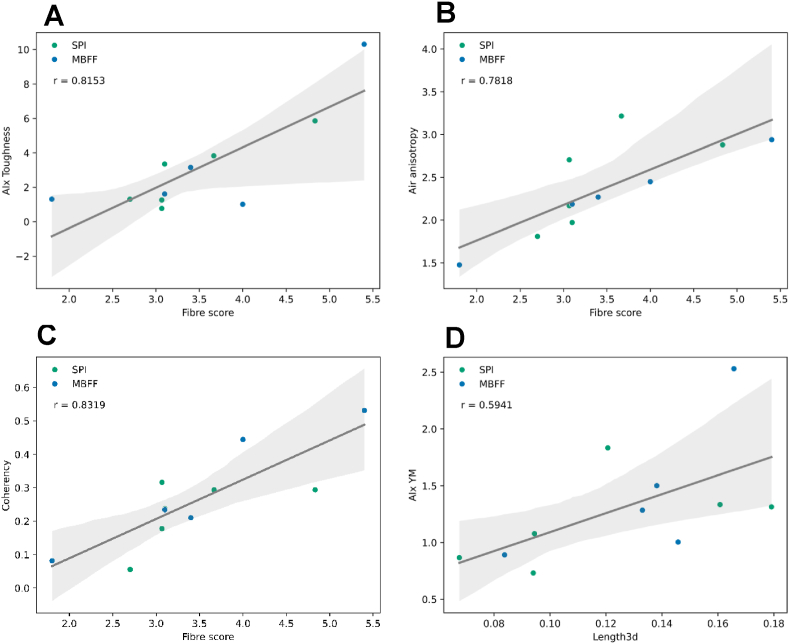
Correlations between A) mechanical properties and macrostructure, B) air bubble microstructure and macrostructure, C) microstructure and macrostructure, and D) mechanical properties and microstructure. Blue datapoints correspond to samples from the MBFF product set and green datapoints correspond to samples from the SPI product set. Shaded regions represent the 95% confidence interval.

While a discussion of all correlations is outside the scope of this manuscript, we selected four product-set independent correlations from Fig. 1 to describe in more detail as argued below. One notably strong correlation is between fibre score and the toughness anisotropy index (r = 0.8153). High fibre scores are related to a higher anisotropy index for toughness measured using tensile tests (Fig. 2A). Thus, there is a relationship between mechanical anisotropy and macrostructural anisotropy. High mechanical anisotropy refers to a weaker structure in one direction compared to another. When the macrostructure of the sample is exposed for macrostructural analysis, weaker samples are likely to fracture in more than one location, exposing more fibres and resulting in a higher fibre score or macrostructural anisotropy. There have been several studies previously describing the relation between a fibrous structure on macroscale and mechanical anisotropy, however these were all based on qualitative description of the macrostructure instead of quantitative analysis (Dekkers et al., 2016; Grabowska et al., 2016; Wittek et al., 2021a). As far as the authors know, this is the first study to demonstrate the relationship between mechanical properties and quantitative macrostructural anisotropy of meat analogues. Another study showed that qualitative descriptions of fibrousness at macroscale are not always reflected in mechanical anisotropy in the case of meat analogues produced from pea protein isolate and biopolymers (Schreuders et al., 2022). This discrepancy suggests that qualitative descriptions may fail to identify macrostructural features accurately, or alternatively, the relationship between macrostructure and mechanical properties is product-set dependent.

Next to correlations between mechanical properties and macrostructure, there are also correlations between structure at different length scales. For example, there is a correlation of r = 0.7818 between fibre score and air anisotropy (Fig. 2B), indicating a relationship between properties at macroscale and properties at microscale. More specifically, more elongated air bubbles at microscale corresponded to a higher fibre score at macroscale. This finding also helps to explain the macrostructure improvement previously found upon gas injection, also known as micro-foaming, during high moisture extrusion cooking (Ghanghas et al., 2024; Zink et al., 2023). Air bubbles are not the only relevant microstructural parameter that correlated with macrostructure. A positive correlation between coherency, describing how much of the sample aligns with a dominant direction on microscale, with fibre score is found as well (Fig. 2C). This means that meat analogues with a higher coherency, indicating a greater alignment of the material in a specific direction, tend to have higher fibre scores, suggesting a more pronounced fibrous structure at macroscale. Previous research on microstructure of pea protein extrudates found similar coherency values as in this study, but did not find any relations with mechanical properties (Gaber et al., 2023). In this study, coherency and mechanical properties are clearly linked as evidenced by several positive and negative correlations (Fig. 1).

Besides coherency, the air bubble microstructural parameters are also related to mechanical properties of the material. There is a positive correlation between the anisotropy index of the Young's Modulus and air bubble length (Fig. 2D) suggesting that the length of air bubbles may affect mechanical performance of a material. The clear relation between air bubble length and elastic behaviour has been well-established in non-food materials. For example, the elastic moduli in the parallel direction were found to increase with a higher air bubble aspect ratio, while the elastic moduli in the perpendicular direction decreased with a higher air bubble aspect ratio (Chao et al., 1999). In the context of mechanical anisotropy, previous studies on food have primarily reported anisotropy indices derived from fracture parameters measured at large deformation (Dekkers et al., 2016; Singh et al., 2024; Snel et al., 2023; Wittek et al., 2021a). This is because large deformation properties are more influenced by the presence of weaker and stronger areas in the sample than small deformation properties (van Vliet, 1996). However, we emphasize that the anisotropy index of the Young's Modulus also provides valuable insights in accordance with a prior study showing the high anisotropy index of the Young's Modulus in cooked chicken meat, which was not found in meat analogues (Schreuders et al., 2019). This difference in anisotropy could be an explanation why consumers perceive differences in texture between meat and meat analogues. This further highlights that when attempting to create meat analogues that mimic real meat, the anisotropy of the Young's Modulus may be a relevant parameter. The relationship between microstructure and mechanical properties opens the possibility for making design rules for specific mechanical performance of a material by setting the air bubble length. Another interesting trend for all correlations in Fig. 2 is the higher confidence interval at higher measured values. This suggests that the more heterogeneous the structure becomes, the less predictable the structure is, which is by itself an inherent property of heterogeneous materials. In a way, the higher confidence interval at higher measured values could be a measure of structure as well.

### 3.2 Contribution of parameter variance to combined product sets

**Fig. 3:**
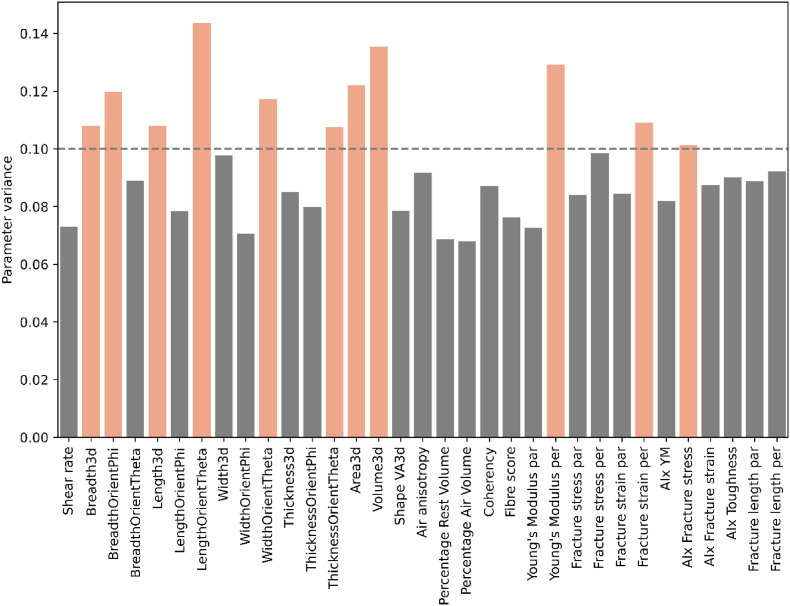
Contribution of parameter variance of the different parameters in combined product sets. Coloured bars indicate parameters with a parameter variance higher than 0.10. AIx refers to anisotropy index, par refers to measured parallel to the shear flow, per refers to measured perpendicular to the shear flow.

The parameter variance can serve as a valuable tool for studying the importance of those parameters within the combined product sets. Parameter variance in this study is defined as the variance between products, not among one product or between replicates. In this analysis, we incorporated new parameters with only a single measurement, including the standard deviations of mechanical properties, the decrease in Poisson's ratio (calculated from the DIC), and the heterogeneity in strain distribution parameters (derived from the fracture strain distribution from DIC). Generally, parameters with high parameter variance are expected to capture diverse patterns or spread in the dataset and could potentially be useful in for example predictive modelling. Parameters with a low parameter variance are parameters that do not change much between products and thus indicate more stability or robustness but may be less relevant for distinguishing between products. The parameters exhibiting high parameter variance across the combined product sets included Breadth3d, Length3d, WidthOrientTheta, ThicknessOrientTheta, LengthOrientTheta, Area3d, Volume3d, Young's Modulus per, Fracture strain per, AIx fracture stress, the standard deviation of the Young's Modulus in both directions, fracture strain in both directions, fracture stress in parallel direction, fracture length in parallel direction and the heterogeneity in strain distribution parameters (Fig. 3).

Interestingly, the highlighted parameters from the parameter variance analysis are mostly different than those showing strong correlations in the correlation matrix (Fig. 1, Fig. 3). Correlation matrices are linear relationships, so it is possible that certain parameters have non-linear relationships and thus do not correlate, yet still have a high parameter variance. Another explanation is that the individual parameters with high parameter variance provide unique information about the product sets, even if they do not correlate with other parameters. One such parameter is the anisotropy index of the heterogeneity in strain distribution, as this parameter did not show any strong correlations with other parameters (data not shown). This parameter describes the anisotropy index of the strain distribution upon fracturing of the product. A high parameter variance of this parameter suggests that it effectively describes differences between products. Previous research already qualitatively showed that strain distribution differed between isotropic and anisotropic meat analogues (Schlangen et al., 2023c) and between foods 3D printed at different infill angles (Ma et al., 2024b). The lack of correlations between strain distribution and other parameters in the product sets indicates that strain distribution upon fracture is not well captured by another mechanical parameter, nor by macro- and microstructure. Nevertheless, this parameter is specifically interesting because it describes the distribution of local strain, unlike most mechanical tests that measure averaged, bulk properties of a material. Last, it is important to note that parameters with lower parameter variance may still provide useful information when considered alongside other parameters, for example when they show high correlations (Fig. 1). For instance, although the fibre score had a relatively low parameter variance, it showed strong correlations with microstructural and mechanical parameters.

### 3.3 Product-set dependent correlations

**Fig. 4:**
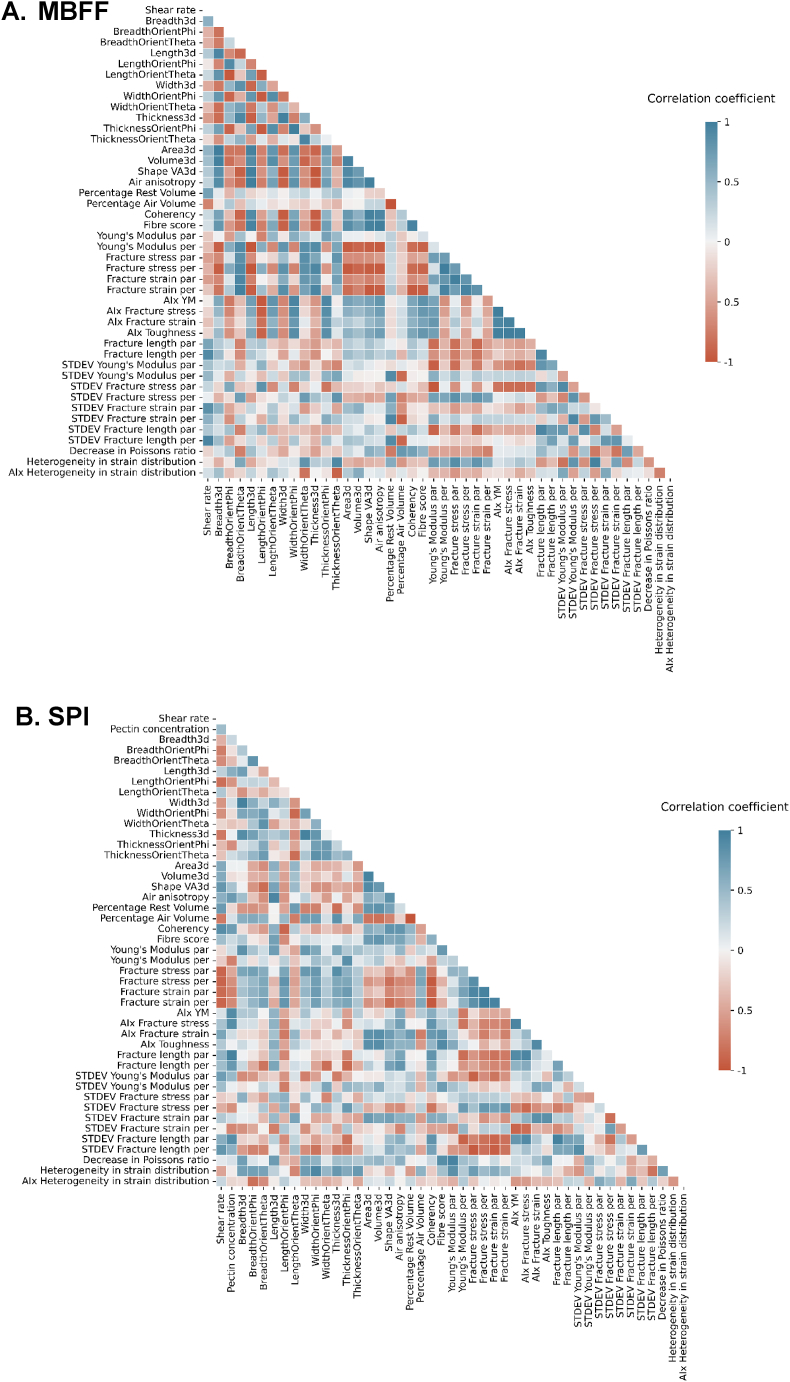
A) Correlation matrix of MBFF product set based on averages and B) correlation matrix of SPI product set based on averages. AIx refers to anisotropy index, par refers to measured in the parallel direction to the shear flow, and per refers to measured in the perpendicular direction to the shear flow.

The previous sections have shown the product-set independent correlations, which describe universal relationships across the products. Certain relationships may not be universal, but are instead unique to a certain product set. By splitting the dataset according to ingredient and making a separate correlation table for the samples made from mung bean fine fraction (MBFF) and soy protein isolate (SPI) product-set dependent correlations arise (Fig. 4). In general, the correlation matrices reveal that the product-set dependent correlations are stronger than the correlations in the combined correlation matrix (Fig. 1). This suggests the presence of product-set dependent correlations and/or that some correlations, potentially product-set independent, will not align in the same order of magnitude (Simpson's paradox). The fact that there are product-set dependent correlations indicates that some relationships between mechanical and structural parameters are influenced by composition or specific architecture of the product. MBFF and SPI likely have different mechanisms of fibre formation, due to for example differences in protein and/or ingredient composition and functional properties like gelling and water holding capacity (Kyriakopoulou et al., 2021; Richter et al., 2024; Zink et al., 2024). Thus each product-set has unique, inherent micro- or nano-structural characteristics, leading to non-correlatable relationships with mechanical and macrostructural parameters. As such this is well known in food applications. For example, when making bread, it is important to choose wheat flour as starting materials, since other raw materials are not able to create this product.

MBFF products exhibited a strong positive correlation between decrease in Poisson's ratio and percentage air volume (Fig. 4A). The more air in the product, the higher the decrease in Poisson's ratio, as the strain in the transversal direction during tensile testing decreases more rapidly with increased air content in the product. Relationships between Poisson's ratio and porosity have been previously linked to pore shape. Poisson's ratio moves towards a critical value of 0.2 for spherical pores and 0–0.2 for elliptical pores and thin cracks upon increasing porosity (Lutz and Zimmerman, 2021). Furthermore, synthetic auxetic materials also experience a decrease in Poisson's ratio under mild strain conditions which was explained by transformations of the air bubble shapes (Bertoldi et al., 2010).

In general, fibre score is an important parameter in the MBFF product set, demonstrating a strong relationship between macrostructure, microstructure, and mechanical properties (Fig. 4A). For MBFF, fibre score correlates with most anisotropy indices of mechanical properties. Notably, fibre score correlated positively with the anisotropy index of the Young's modulus. This suggests that bigger differences in stiffness between parallel and perpendicular samples in MBFF are reflected in the macrostructure of the products.

In the MBFF product set, mechanical anisotropy indices show clustering, suggesting they may contain the same information (Fig. 4A). Interestingly, this indicates that mechanical anisotropy occurs throughout the entirety of the tensile tests for MBFF product set, in both the linear regime and the non-linear regimes. This is unique behaviour, as for most meat analogue materials anisotropy occurs primarily in the non-linear regime (Taghian Dinani et al., 2023).

The independent variable, shear rate, in the MBFF product set also correlated to microstructural and mechanical parameters (Fig. 4A). Shear rate correlated positively with the fracture length in the perpendicular direction (Fig. 4A). Increased shear rate resulted in a longer fracture length perpendicular to the shearing direction, indicating that shearing delayed macrocracking in the meat analogue. Homogeneous materials tend to nucleate fracture less and fracture rather abrupt, resulting in a short fracture length, while heterogeneous materials tend to fracture in multiple stages, delaying the transition from microcrack to macrocrack depending on the material structure (van Mier, 1997). Additionally, one of the strongest negative correlations is between shear rate and percentage of air volume; higher shear rates corresponded to lower percentages of air volume. This suggests that shearing decreases the ability of the continuous phase to retain air bubbles in the product. High shear rates, similar to intensive mixing, may cause air bubbles to break up into smaller air bubbles that disperse throughout the product, potentially falling below XRT resolution of 6 μm. Thus, increasing the shear rate alters the macro- and microstructure, making the material more heterogeneous and extending the fracture length during tensile elongation in products based on MBFF and processed in the shear cell.

By contrast, certain correlations are unique to the SPI product set and do not appear in the MBFF product set. Some of these correlations are related to the independent variable, pectin concentration, for example - pectin concentration is found to correlate positively with air anisotropy (Fig. 4B). Previous research showed that the complex modulus of a blend of SPI and pectin was higher than that of SPI alone (Dekkers et al., 2018). Increasing the pectin concentration in the SPI blend probably enhances local elasticity and prevents elongated air bubbles from reverting to their original shape. Furthermore, pectin concentration is negatively correlated to most of the mechanical properties measured in both the parallel and the perpendicular direction (Fig. 4B). This aligns with the finding that air anisotropy is negatively correlated with many of the same mechanical properties. It is therefore likely that pectin weakens the mechanical performance of the product by increasing local elasticity and facilitating air bubble elongation. Furthermore, the addition of pectin makes the material fracture more gradually, as indicated by the positive correlation between pectin concentration and fracture length in the direction parallel to the shear flow (Fig. 4B).

Unlike for the MBFF product set, in the SPI product set the AIx of the mechanical properties do not cluster together but behave differently. For instance, the anisotropy indices of the fracture strain and toughness show positive correlations with fibre score, while fibre score does not correlate with the anisotropy indices of the Young's Modulus and fracture stress (Fig. 4B). Furthermore, the SPI product set reveals positive correlations between the percentage of air volume and the width and thickness of the air bubbles, while showing negative correlations with the surface area (Area3d) and volume of the air bubbles (Volume3d) (Fig. 4B). Therefore, a higher percentage of air volume indicates smaller air bubbles in the case of the SPI meat analogues in this study. Previous research has shown that smaller air bubbles are less deformed than larger ones in shear cell meat analogue products, which explains the observed increase in width and thickness (Snel et al., 2024; Wang et al., 2019).

Overall, the percentage of air volume and the percentage of rest volume are more strongly correlated with other parameters in the SPI product set compared to the MBFF product set. In the case of SPI, these percentages correlated strongly with the anisotropy index of fracture strain and toughness (Fig. 4B). The MBFF product set appears to show many correlations with fibre score, while the SPI product set shows many correlations with percentage of air volume. Thus, we can argue that macrostructure is dominating in the MBFF product set, while microstructure may be dominating in the SPI product set. These differences between the MBFF product set and the SPI product set reveal structural information. More specifically, if the SPI-based products are characterized by very small air bubbles or a dense protein network, it becomes evident that microstructure significantly influences measurements performed at microscale.

### 3.4 Contribution of parameter variance to the separate product sets

**Fig. 5:**
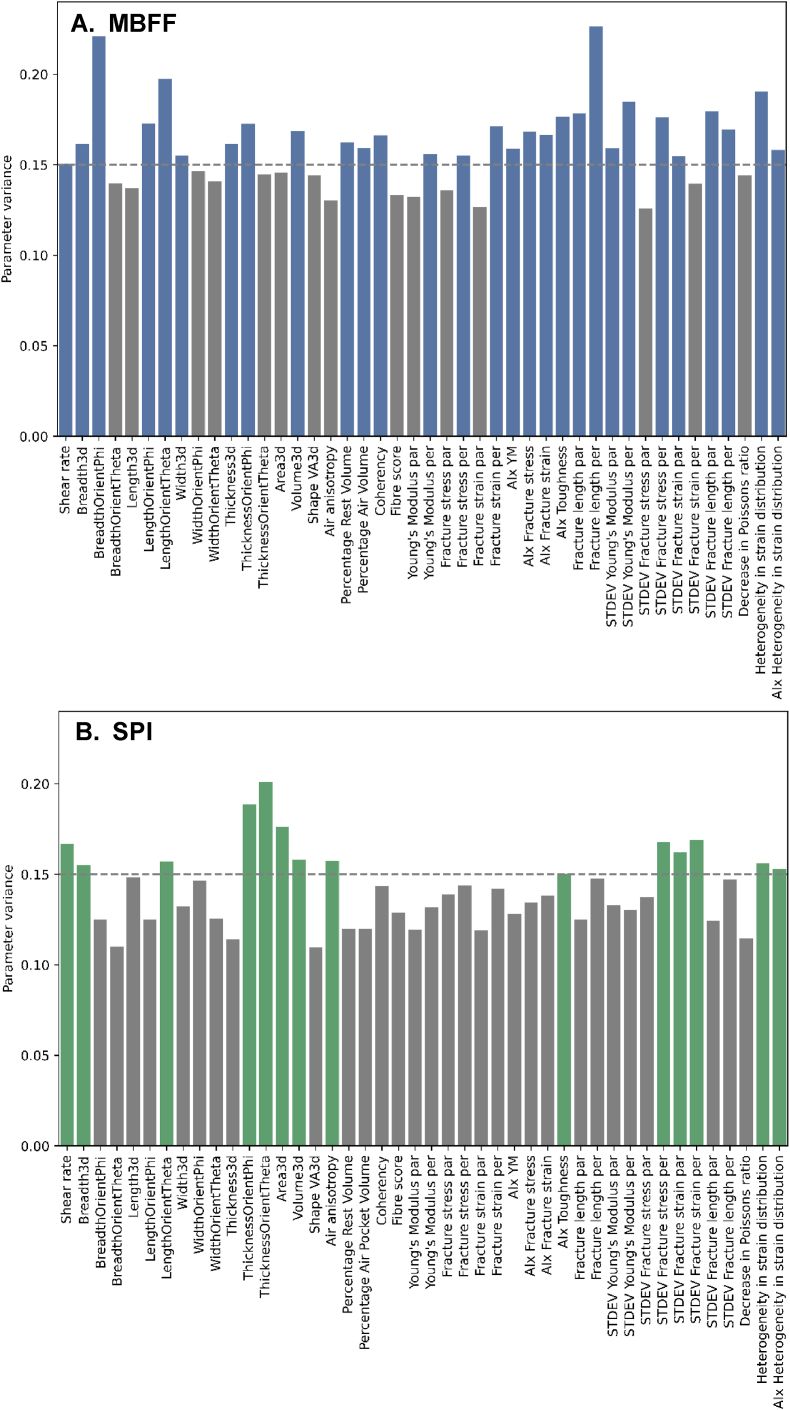
Contribution of variance of the different parameters in the product set of A) MBFF and B) SPI. Coloured bars indicate parameters with a variance higher than 0.15.

The parameter variance contribution in the separate product sets is studied for MBFF and SPI individually (Fig. 5). Generally, the parameter variance in the separate product sets is higher than in both product sets combined (Fig. 3, Fig. 5); the parameter variance threshold is set higher to 0.15 for the separate product sets instead of 0.10 as in the product sets combined. Combining the product sets blurs out differences between products, reducing the parameter variance. MBFF contained more high variance parameters than SPI for the same parameter variable threshold as seen in Fig. 5A and B. Interestingly, in both product sets the parameters on heterogeneity of strain distribution upon tensile elongation are high in parameter variance, similar to the combined parameter variance plot (Fig. 3). This observation stresses the importance of the strain distribution parameters in visualizing a spread in the product sets again.

While fibre score showed strong correlations in the MBFF product set (Fig. 4A), the contribution of variance is relatively low (Fig. 5A) in a range that was similar to the SPI product set (Fig. 5B). Furthermore, the percentage of rest volume and percentage of air volume are not found to be very dominant in the correlation matrix, yet did exhibit high variance in the MBFF product set (Fig. 5A). Additionally, the fracture length and all the mechanical anisotropy parameters show high variance, suggesting that specifically these parameters can successfully describe differences between samples within the product set.

In the SPI product set, percentage of air volume and rest volume showed strong correlations with various parameters (Fig. 4B), but their variance contribution is relatively low (Fig. 5B). Furthermore, compared to MBFF, the contribution of mechanical parameters to the variance is relatively low in SPI. However, air anisotropy stood out with high variance in the SPI product set (Fig. 5B).

### 3.5 Microstructural differences from XRT and CLSM

Currently, microstructural parameters have been derived mostly from XRT analysis, which quantifies the air bubble morphology in the meat analogues, yet sample microstructure can also be studied with microscopy, such as CLSM. This section describes correlations between microstructural parameters from both techniques to determine if air bubbles are the primary feature of importance for the microstructure and to compare the two methods. Staining with Rhodamine B highlighted the protein parts of the samples in CLSM. The images reveal that most of the area is protein, with some non-protein domains (Supplementary Materials S3). The addition of pectin in the case of SPI and the increase of shear rate in the case of MBFF changed the microstructure of the materials, specifically the size and shape of the non-protein domains.

**Fig. 6:**
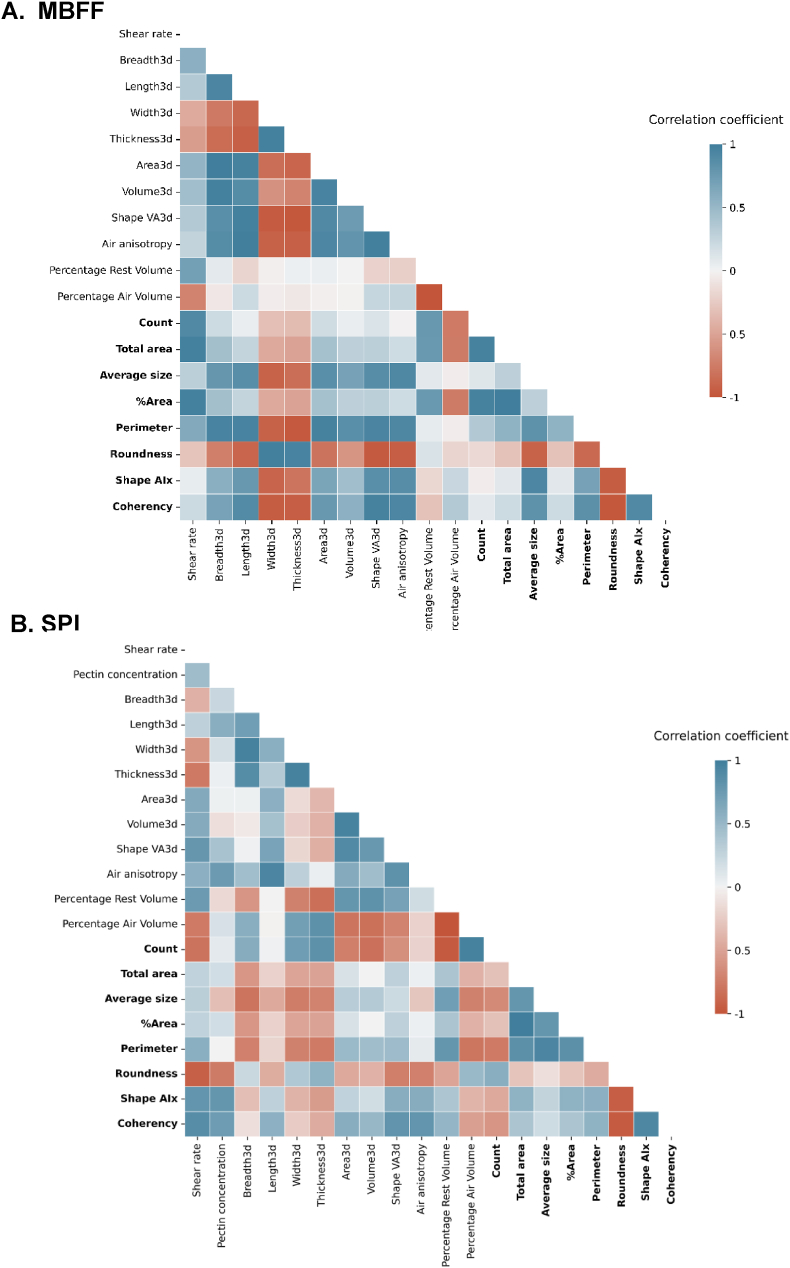
A) correlation matrix of XRT parameters and CLSM parameters for the MBFF product set, B) highlighted correlation between macrostructure and microstructure for the MBFF product set, C) correlation matrix of XRT parameters and CLSM parameters for the SPI product set, and D) highlighted correlation between mechanical properties and microstructure for the MBFF product set.

Fig. 6A and B shows the correlation matrices for the microstructural parameters extracted from XRT and the microstructural parameters extracted from CLSM (in bold) for the MBFF product set and the SPI product set. Both product sets exhibit a strong correlation between air anisotropy measured using XRT and anisotropy index (Shape AIx) measured using CLSM (Fig. 6A and B). This suggests that the non-protein domains that were identified and analyzed using microscopy are likely air bubbles, consistent with past research (Snel et al., 2024).

**Fig. 7:**
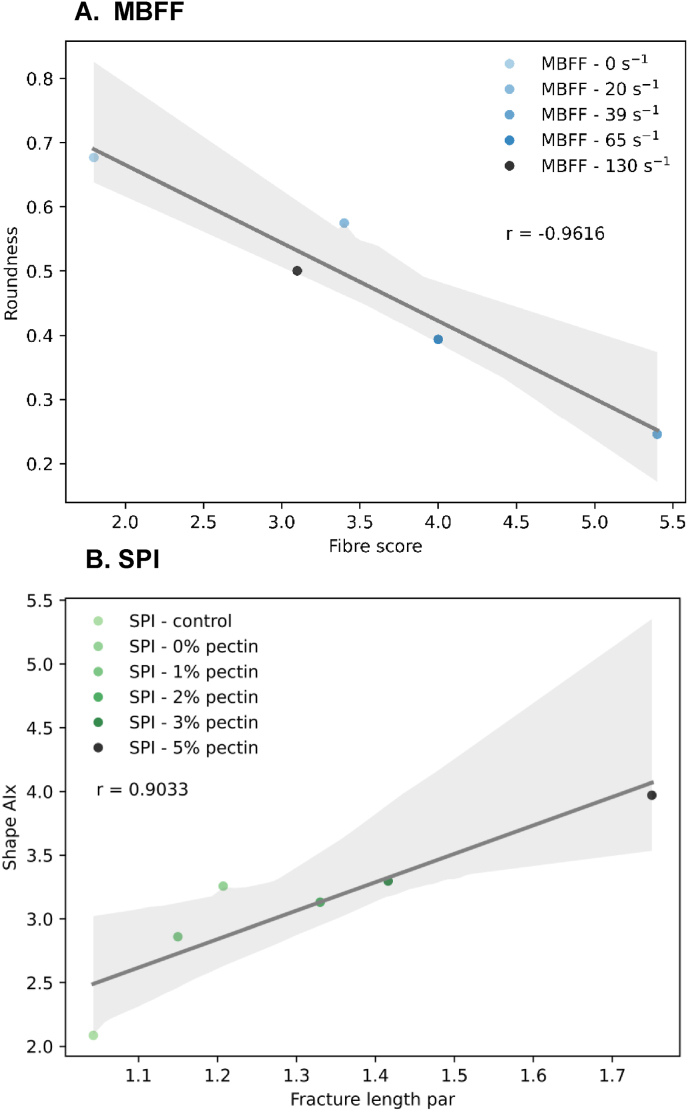
A) Microstructural parameter from CLSM analysis correlating with macrostructural parameter for MBFF product set, and B) Microstructural parameter from CLSM analysis correlating with mechanical parameter for SPI product set. Shaded regions represent the 95% confidence interval.

Additionally, correlations are observed between microstructure as measured with CLSM and macrostructure as measured with Fiberlyzer. In the MBFF product set, a decrease in roundness parameter of the identified air bubble shapes in CLSM correlate positively with an increase in fibre score from Fiberlyzer (Fig. 7A), reflecting similar behaviour as in Fig. 4B. When air bubbles become more elliptical, the macrostructure of the meat analogues tends to become more fibrous.

In the SPI product set, the correlation between air anisotropy from XRT and anisotropy index (Shape AIx) from CLSM is relatively weak, as air bubble morphology is not as well captured in 2D (CLSM) compared to 3D (XRT). An interesting correlation to highlight in the SPI product set is between the anisotropy index (Shape AIx) from CLSM and the mechanical parameter fracture length in the parallel direction (Fig. 7B). This strong positive correlation suggests that more elongated air bubbles at microscale extend the fracture length during tensile testing, indicating that the shape and orientation of air bubbles likely influences the mechanical performance of the products.

In both the MBFF and the SPI product sets, the coherency, the extent to which the microstructure aligns in a dominant direction, was strongly positively correlated to the anisotropy index as measured with CLSM (Fig. 6A and B). This is not surprising, as the dominant direction in the meat analogues is likely formed by the shear direction during processing, much like how the elongation of air bubbles occurs. This highlights that air bubble morphology is likely the most important microstructural parameter for the product sets examined in this study.

### 3.6 Univariate feature selection

Univariate feature selection can be used to explore which parameters are most important for a specific target feature. Univariate feature selection is performed on the product sets combined, the MBFF product set, and the SPI product set. To perform univariate feature selection, a target feature is needed. Ideally, we would choose “texture” or “fibrousness” as a whole as the target, but since these terms are not well-defined, we opt for representative features that theoretically describe anisotropy for each measurement technique. More specifically, AIx Toughness is selected for mechanical properties, fibre score is selected for macrostructure, and air anisotropy is selected for microstructure (XRT).

Table 2 summarizes the top three selected features based on univariate feature selection for a specific target feature. When using fibre score as the target, microstructure features consistently are found important, regardless of the experimental product set. This finding strongly suggests a relationship between macrostructure and microstructure, independent of the experimental product set. For the target feature of AIx Toughness, the selected features vary depending on the experimental product set used. In the MBFF product set, only mechanical parameters are selected, whereas in the SPI product set, mechanical, microstructural and macrostructural parameters are selected. For the target feature of air anisotropy, the selected features from the product sets combined included both structural features at microstructure and macrostructure, along with a mechanical parameter. This is a strong indication that microstructural differences affect macrostructure as well as mechanical properties. However, in the case of the SPI product set, the selected features for the target feature of air anisotropy did not include fibre score, suggesting a potentially weaker connection between the two compared to the MBFF product set.

**Table 2:** The three selected features from univariate feature selection of target features: fibre score, AIx Toughness and air anisotropy.

Product set	Target feature	Selected features
Combined	Fibre score	BreadthOrientTheta, Air anisotropy, Coherency
	AIx Toughness	Area3d, AIx YM, AIx Fracture stress
	Air anisotropy	Fibre score, Fracture strain per, Coherency
MBFF	Fibre score	Air anisotropy, Coherency, AIx YM
	AIx Toughness	AIx YM, AIx Fracture stress, AIx Fracture strain
	Air anisotropy	Fibre score, Young's Modulus per, Fracture stress per
SPI	Fibre score	Length3d, WidthOrientTheta, LengthOrientPhi
	AIx Toughness	Length3d, Fibre score, STDEV Fracture strain par
	Air anisotropy	Length3d, Shape VA3d, Fracture length per

From a broader perspective, depending on the scope of the study, one can select a target feature and explore which other features behave similarly. For example, this study demonstrates that the fibre score measurement using Fiberlyzer, a relatively simple, accessible, and cheap technique, can also provide an indication about the behaviour of microstructural properties, particularly the air bubble microstructure. Measurement of the anisotropy index of the toughness further provides information on much of the mechanical behaviour of the material. These two measurements may suffice for general characterization, such as for characterizing variation of ingredients and processing conditions. However, for detailed understanding of material properties and structure formation, additional parameters are highly valuable. In particular strain distribution from DIC analysis may provide unique information due to its high parameter variance but relatively weak correlations with other parameters.

## 4 Conclusions

This paper has established several relationships between measurable structural quantities of eleven different products varying in composition within two different meat analogue product sets based on soy and mung bean respectively. We have shown that anisotropy-related parameters, such as fibre score, air anisotropy and the toughness anisotropy index show the strongest positive correlations with each other. Despite the differences between the product-sets, correlations are observed across different length scales and structural characteristics are reflected in mechanical properties. These correlations thus show fundamental relationships that hold for all meat analogues in this study irrespective of their composition or processing conditions. Interestingly, several correlations are not universal, but product-set dependent. For instance, in the MBFF product set, the relationship between macrostructure and microstructure is more pronounced, with fibre score playing a more important role. By contrast, the total air volume at microstructure is found more important in the SPI product set. These product-set dependent correlations can provide valuable insights into the distinct ways that structure architecture is formed across different products, stressing roles of specific ingredients and processing conditions. The XRT and CLSM analyses show microstructural similarities, confirming that air bubbles are the main microstructural parameter of importance. Although heterogeneity in strain distribution does not show many correlations in the correlation matrix, they did exhibit high variance independent of the product set and might therefore provide additional insights in meat analogue structure. Further research could study meat analogues produced from a range of different ingredients to determine if the product-set independent correlations are consistent across these products. The next step is to connect these results to sensory tests to determine which parameters correlate with fibrousness and texture as perceived by consumers.

## CRediT authorship contribution statement

**Miek Schlangen:** Conceptualization, Formal analysis, Writing – original draft, Writing – review & editing, Visualization. **Iris van der Doef:** Investigation, Writing – review & editing. **Atze Jan van der Goot:** Writing – review & editing, Supervision. **Mathias P. Clausen:** Writing – review & editing, Supervision. **Thomas E. Kodger:** Conceptualization, Writing – review & editing, Supervision.

## Data availability

Data underlying this publication are available in the 4TU data repository: https://doi.org/10.4121/f72387bc-25ca-40e9-a6a1-6b2d0b659e30.

## Declaration of competing interest

None.

## Data Availability

The data will be published in a data repository.
