# Supplementary material for: Meat analogues: The relationship between mechanical anisotropy, macrostructure, and microstructure
Source: Curr Res Food Sci. 2025 Jan 21;10:100980. doi: 10.1016/j.crfs.2025.100980 (PMC11836517; doi:10.1016/j.crfs.2025.100980)
Supplement: Multimedia component 1 [file mmc1.docx]

**Supplementary materials**

**S1: Definition of parameters used in this study**

| **Parameter** | **Description** |
| --- | --- |
| Shear rate | Independent variable: shear rate of the high temperature shear cell process |
| Pectin concentration | Independent variable: pectin concentration added to SPI samples |
| Breadth3d | Largest distance (or width) of the object in a direction perpendicular to the maximum Feret diameter (XRT) |
| Breadth Orient Phi | Angle phi of the Breadth diameter over a range of angles (0, +90) (XRT) |
| Breath Orient Theta | Angle theta of the Breadth diameter over a range of angles (-180, +180) (XRT) |
| Length3d | Maximum of the Feret diameters (XRT) |
| Length Orient Phi | Angle phi of the maximum Feret diameter (XRT) |
| Length Orient Theta | Angle theta of the maximum Feret diameter (XRT) |
| Width3d | Minimum of the Feret diameters (XRT) |
| Width Orient Phi | Angle phi of the minimum Feret diameter (XRT) |
| Width Orient Theta | Angle theta of the minimum Feret diameter (XRT) |
| Thickness3d | The Z direction, if Length would be the X direction and Breath would be the Y direction (XRT) |
| Thickness Orient Phi | Angle phi of the Thickness diameter over a range of angles (0, +90) (XRT) |
| Thickness Orient Theta | Angle theta of the Thickness diameter over a range of angles (-180, +180) (XRT) |
| Area3d | 3D surface area of the object (XRT) |
| Volume3d | Volume of the object (number of voxels in the object multiplied by volume of a single voxel) (XRT) |
| Shape | $\frac{{Area}^{3}}{36\times\pi\times{Volume}^{2}}$. Perfect sphere = 1, larger values = less compact objects (XRT) |
| Air anisotropy | Length3d / Width3d (XRT) |
| Percentage rest volume | Volume percentage of sample, defined based on density with XRT (XRT) |
| Percentage air volume | Volume percentage of air, defined based on density with XRT (XRT) |
| Coherency | Microstructural parameter from CLSM images that describes coherency of the structure to a dominant direction. One dominant direction = 1, no dominant direction = 0 (CLSM) |
| Fibre score | Fibrousness at macroscale (Fiberlyzer) |
| Young’s Modulus par | Slope of the stress-strain curve from a tensile test performed parallel to the shearing direction in the HTSC (tensile test) |
| Young’s Modulus per | Slope of the stress-strain curve from a tensile test performed perpendicular to the shearing direction in the HTSC (tensile test) |
| Fracture stress par | Maximum tensile stress from a tensile test performed parallel to the shearing direction in the HTSC (tensile test) |
| Fracture stress per | Maximum tensile stress from a tensile test performed perpendicular to the shearing direction in the HTSC (tensile test) |
| Fracture strain par | Strain at maximum tensile stress from a tensile test performed parallel to the shearing direction in the HTSC (tensile test) |
| Fracture strain per | Strain at maximum tensile stress from a tensile test performed perpendicular to the shearing direction in the HTSC (tensile test) |
| AIx YM | Anisotropy index of the Young’s Modulus parameters: par/per (tensile test) |
| AIx Fracture stress | Anisotropy index of the Fracture stress parameters: par/per (tensile test) |
| AIx Fracture strain | Anisotropy index of the Fracture strain parameters: par/per (tensile test) |
| AIx Toughness | Anisotropy index of the toughness: par/per. Toughness is defined as the area under the stress-strain curve (tensile test) |
| Fracture length par | \|  \| $\frac{\varepsilon_{failure}}{\varepsilon_{fracture}}$ \|  \| \| --- \| --- \| --- \|   where ε_failure_ is the strain at the failure point when the stress has reached 0 and ε_fracture_ is the strain at the fracture point, both measured in the direction parallel to the shearing direction in the HTSC (tensile test) |
| Fracture length per | \|  \| $\frac{\varepsilon_{failure}}{\varepsilon_{fracture}}$ \|  \| \| --- \| --- \| --- \|   where ε_failure_ is the strain at the failure point when the stress has reached 0 and ε_fracture_ is the strain at the fracture point, both measured in the direction perpendicular to the shearing direction in the HTSC (tensile test) |
| STDEV Young’s Modulus par | Standard deviation of the Young’s Modulus in the parallel direction (tensile test) |
| STDEV Young’s Modulus per | Standard deviation of the Young’s Modulus in the perpendicular direction (tensile test) |
| STDEV Fracture stress par | Standard deviation of the fracture stress in the parallel direction (tensile test) |
| STDEV Fracture stress per | Standard deviation of the fracture stress in the perpendicular direction (tensile test) |
| STDEV Fracture strain par | Standard deviation of the fracture strain in the parallel direction (tensile test) |
| STDEV Fracture strain per | Standard deviation of the fracture strain in the perpendicular direction (tensile test) |
| STDEV Fracture length par | Standard deviation of the fracture length in the parallel direction (tensile test) |
| STDEV Fracture length per | Standard deviation of the fracture length in the perpendicular direction (tensile test) |
| Decrease in Poisson’s ratio | \|  \| $v_{t=linear}-v_{t=failure}$ \|  \| \| --- \| --- \| --- \|   where *v_t=linear_* is the first Poisson’s ratio recorded through DIC analysis and *v_t=failure_* is the last Poisson’s ratio recorded (just before failure) (tensile test and DIC) |
| Heterogeneity in strain distribution | Heterogeneity of the strain distribution in a tensile specimen just before failure of that specimen. Calculated from the slope of the Global Moran’s I versus neighborhood distance fit. A high value thus describes high heterogeneity, while a low value describes more homogeneous strain distribution (tensile test and DIC) |
| AIx heterogeneity in strain distribution | Heterogeneity in strain distribution parallel to shearing direction / heterogeneity in strain distribution perpendicular to shearing direction (tensile test and DIC) |
| Count | Number of isolated shapes (CLSM) |
| Total area | Total area of isolated shapes (CLSM) |
| Average size | Average size of isolated shapes (CLSM) |
| %Area | The area percentage of the isolated shapes (CLSM) |
| Perimeter | The length of the outside boundary of the isolated shape (CLSM) |
| Roundness | $\frac{4\cdot area}{\pi\cdot{length}^{2}}$, also known as the inverse of the aspect ratio (CLSM) |
| Shape AIx | Anisotropy index of the identified shape defined as Feret diameter / width (CLSM) |

**Supplementary materials S2**

Figure S2 shows the correlations of both product sets based on individual datapoints and thus also includes statistical significance. Several assumptions had to be made to prepare this figure. Some parameters were measured in duplicate, while others were measured in triplicate, complicating the calculation of potential correlations. To generate a third datapoint for the parameters measured in duplicate, an average of the two individual datapoints was used. Furthermore, it was not possible to verify whether repeated measurements using one technique were conducted on the same samples as those using another technique. Broadly, the correlations are consistent between Error: Reference source not found and Figure S2, although the magnitudes of the correlations may differ.


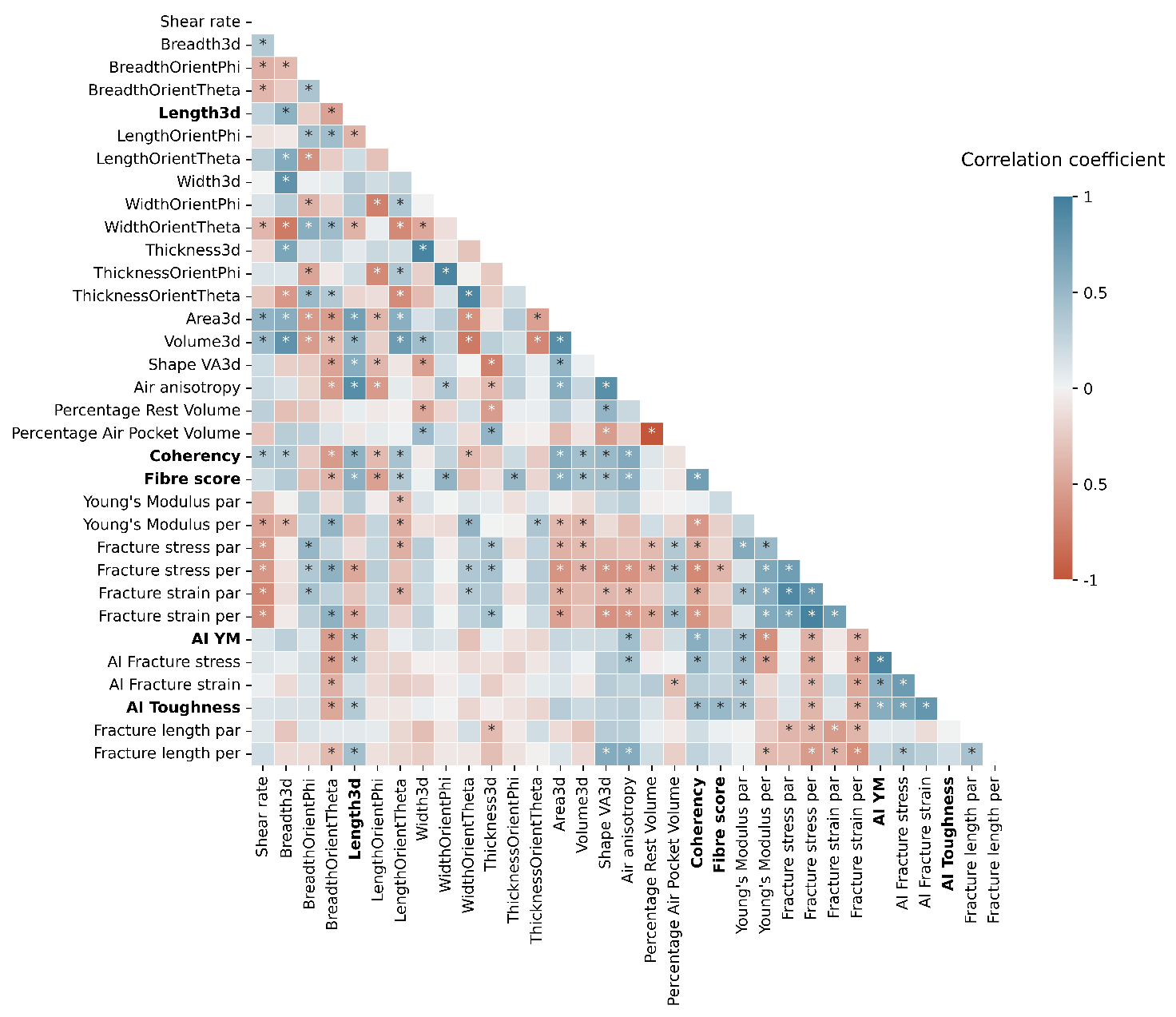


Figure S2 Correlation matrix of entire data including significance. Parameters in bold are further highlighted in Figure 2. AIx refers to anisotropy index, par refers to measured in the parallel direction to the shear flow, and per refers to measured in the perpendicular direction to the shear flow. * refers to a statistical significance of p < 0.05.

**Supplementary materials S3**


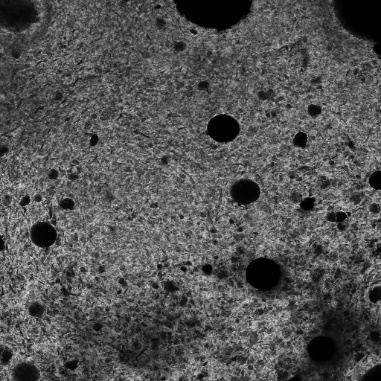

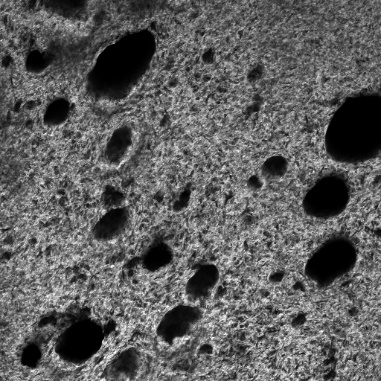

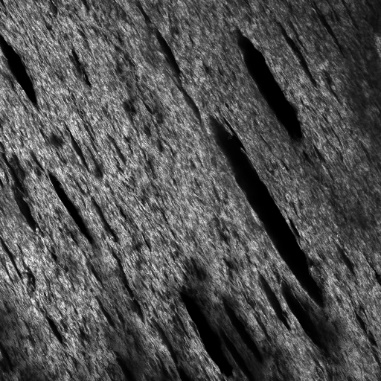


MBFF-0

MBFF-20

MBFF-39


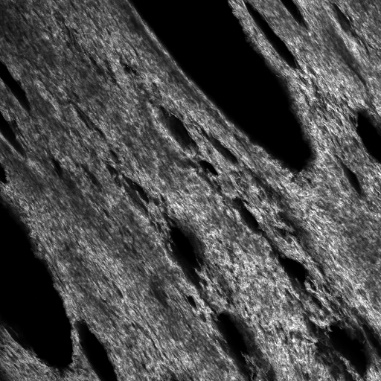

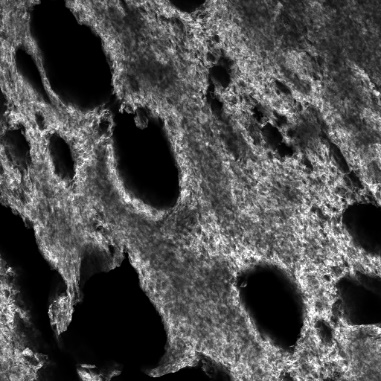


MBFF-65

MBFF-130

Shear direction

Figure S3.1: CLSM images of microstructure of meat analogues produced from mung bean fine fraction at different shear rates of 0, 20, 39, 65, and 130 s^-1^. Scale bar in the top left image represents 200 µm and is applicable to all images.


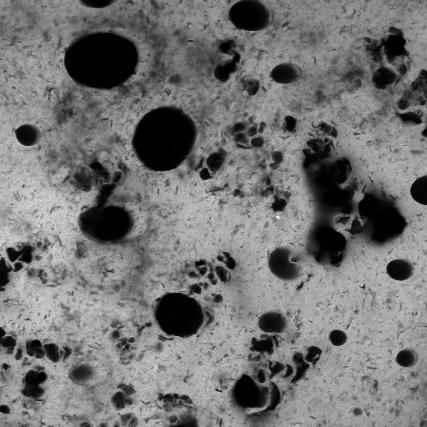

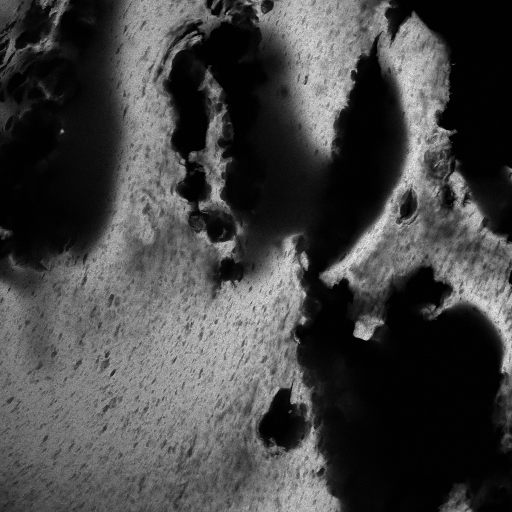

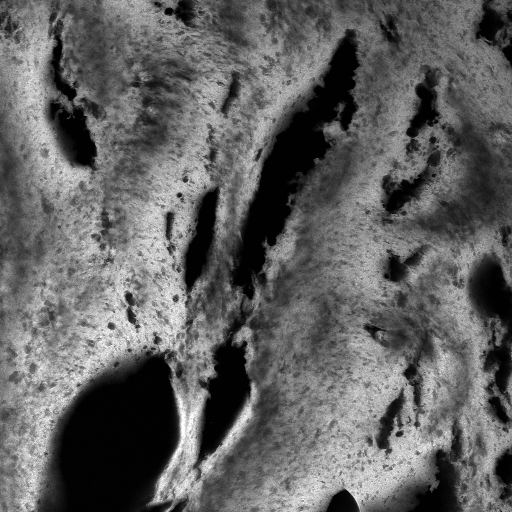

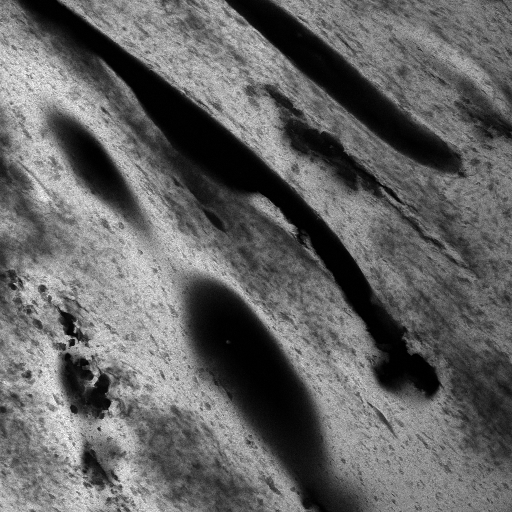

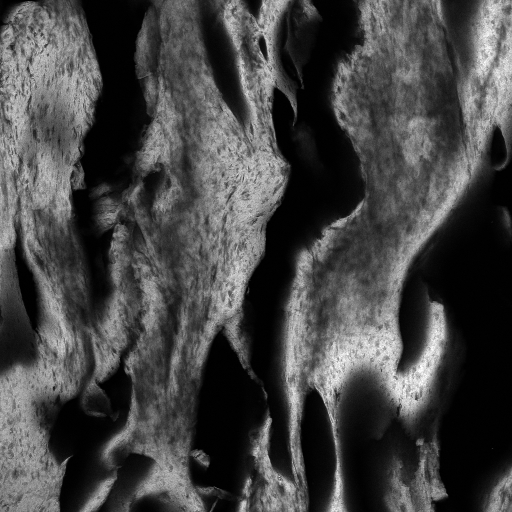

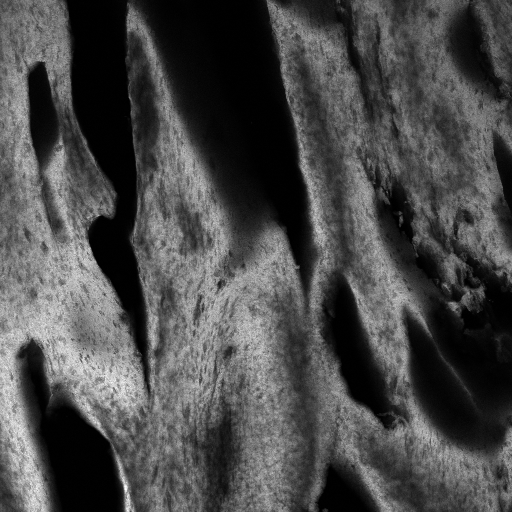


SPI-0-no shear

SPI-0

SPI-1

SPI-2.2

SPI-3.5

SPI-5

Shear direction

Figure S3.2: CLSM images of microstructure of meat analogues produced from soy protein isolate with different concentrations of pectin. The shear direction in the image of SPI-2.2 has been rotated 30 ° to the left. Scale bar in the top left image represents 200 µm and is applicable to all images.

**Supplementary materials S4**

Supplementary material S4 contains additional correlation tables with masks to highlight certain conditions. Correlation was defined here as an absolute correlation coefficient higher than 0.25.

Figure S4.1: Unique correlations for the MBFF product set.


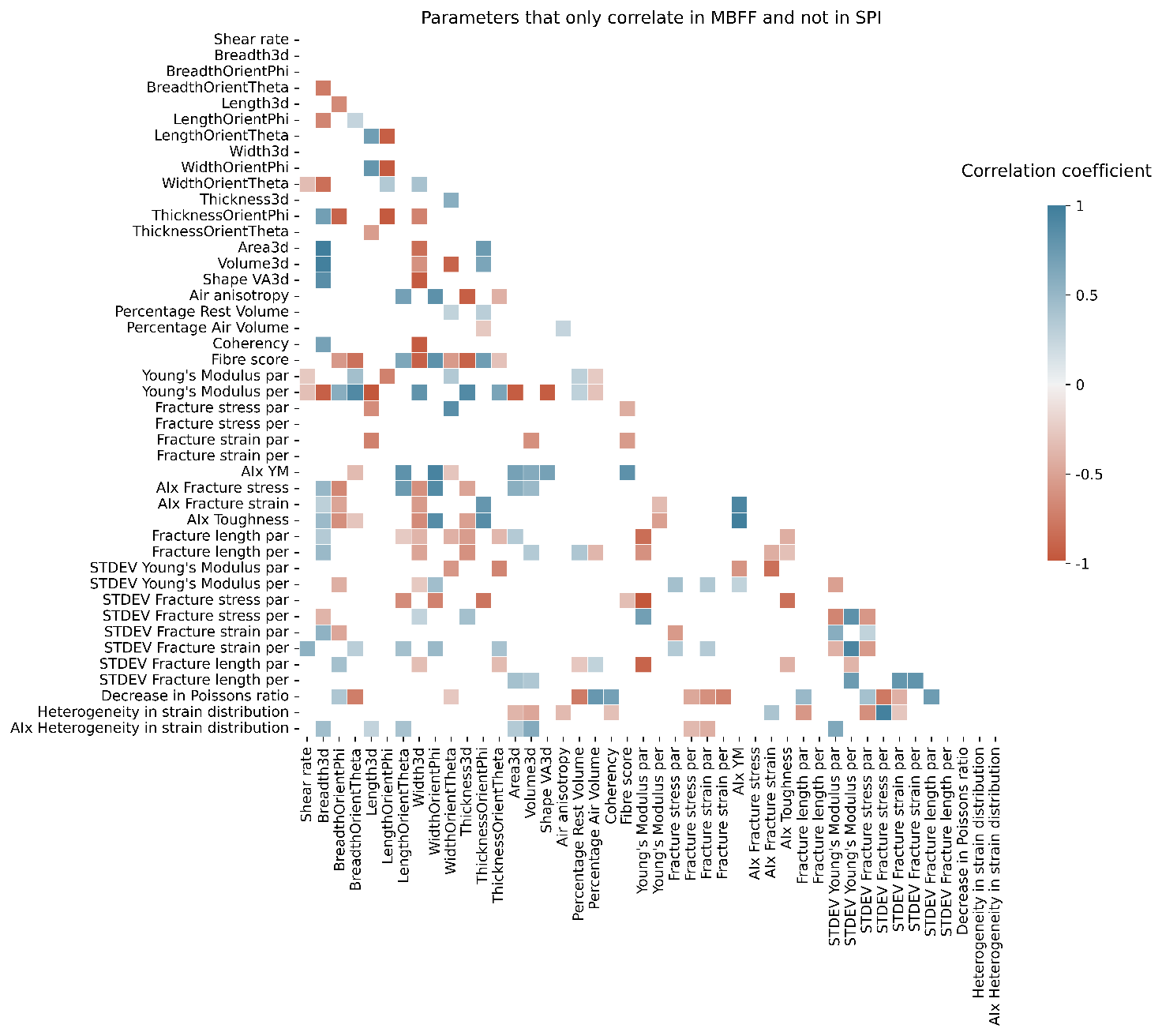


Figure S4.2: Unique correlations for the SPI product set.


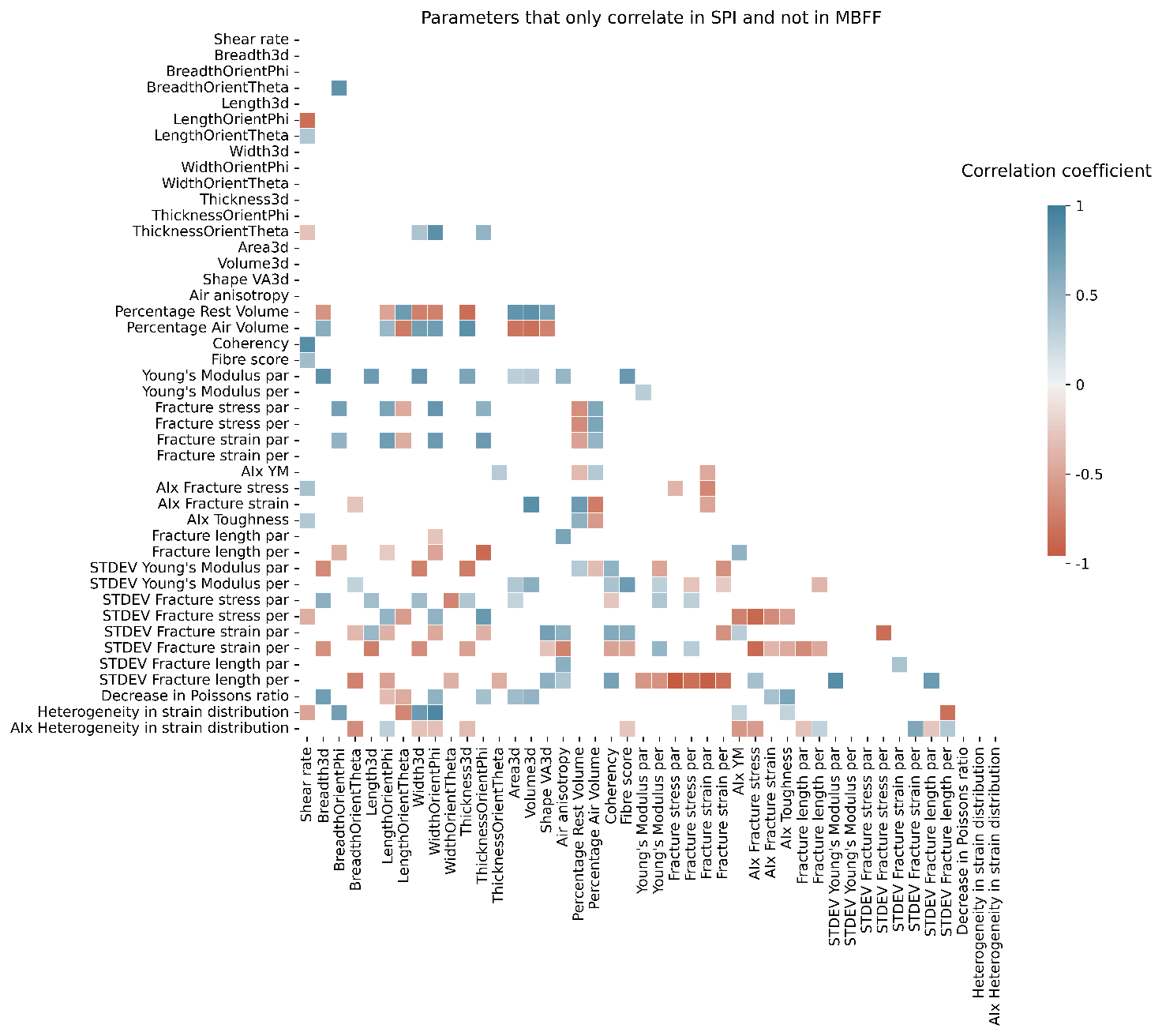


Figure S4.3: Positive correlations in the MBFF product set, which at the same time correlate negatively in the SPI product set.


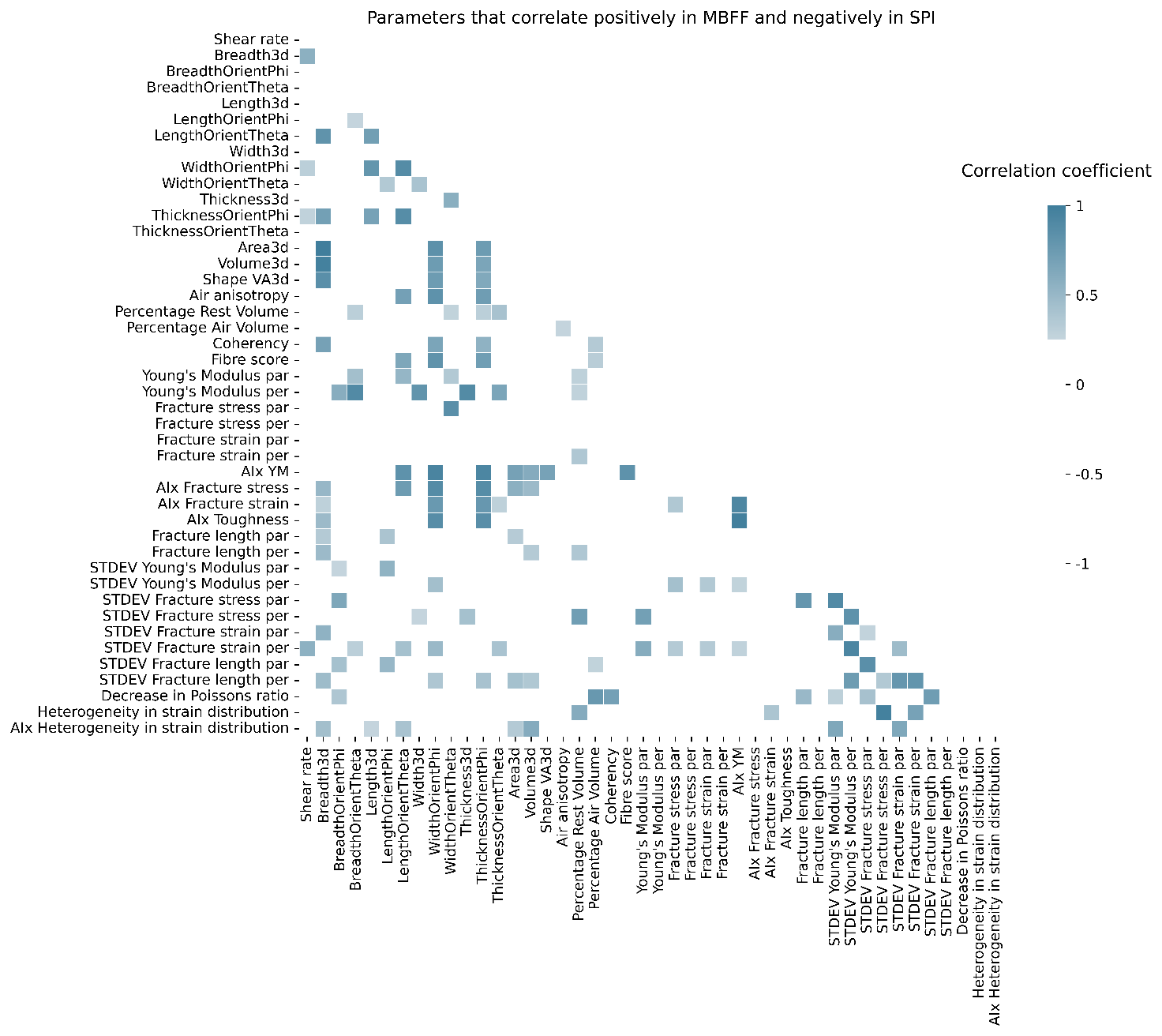


Figure S4.4: Positive correlations in the SPI product set, which at the same time correlate negatively in the MBFF product set.


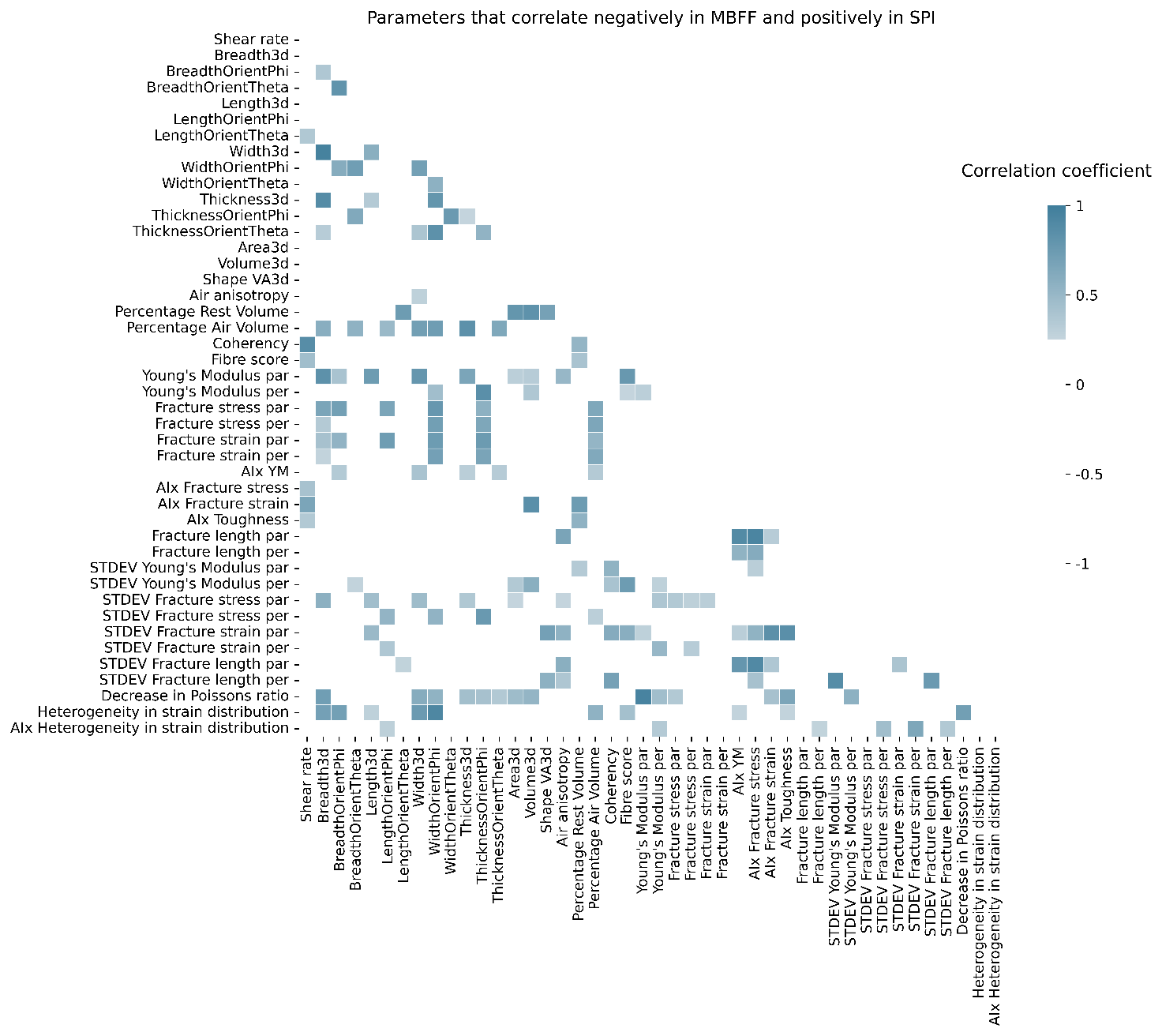


Figure S4.5: Parameters that do not correlate in the MBFF product set nor in the SPI product set.


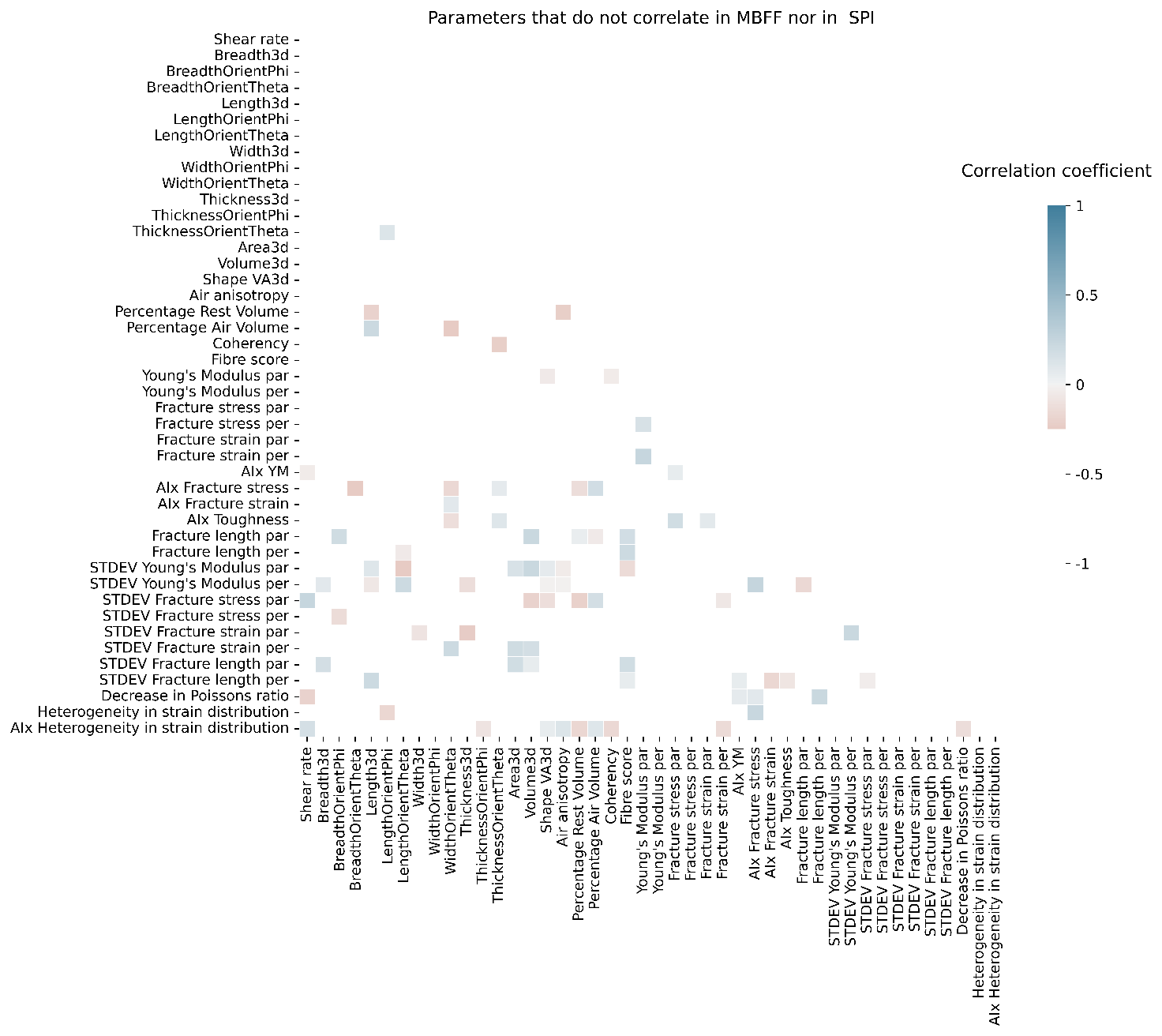


**Supplementary materials S5**

MBFF-0

MBFF-20

MBFF-39

MBFF-65

MBFF-130

Figure S5.1: Macrostructural images of meat analogues folded open to expose the inner structure parallel to the shear direction. Samples are produced from mung bean fine fraction with 0.5% transglutaminase and processed at different shear rates of 0, 20, 39, 65, and 130 s^-1^. Samples have been frozen after production and thawed before macrostructural analysis.


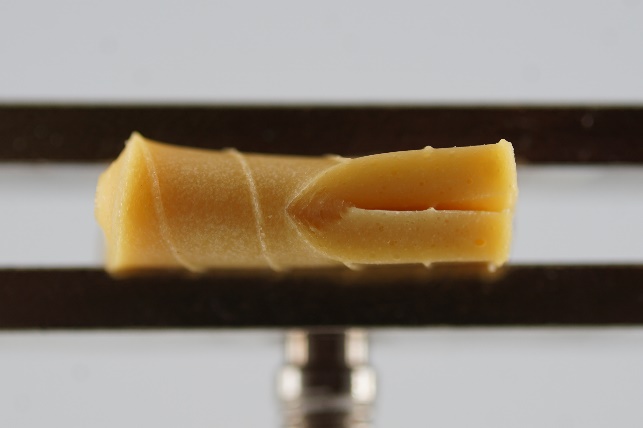

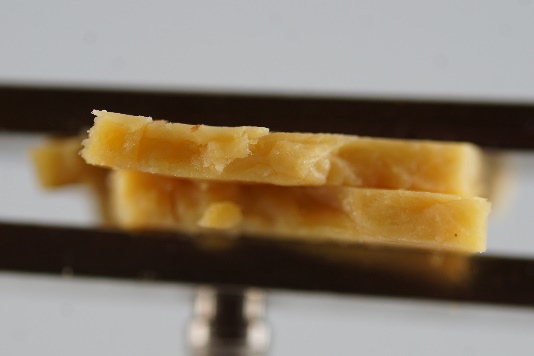

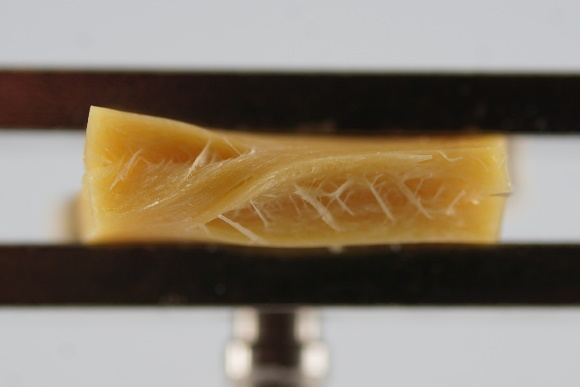

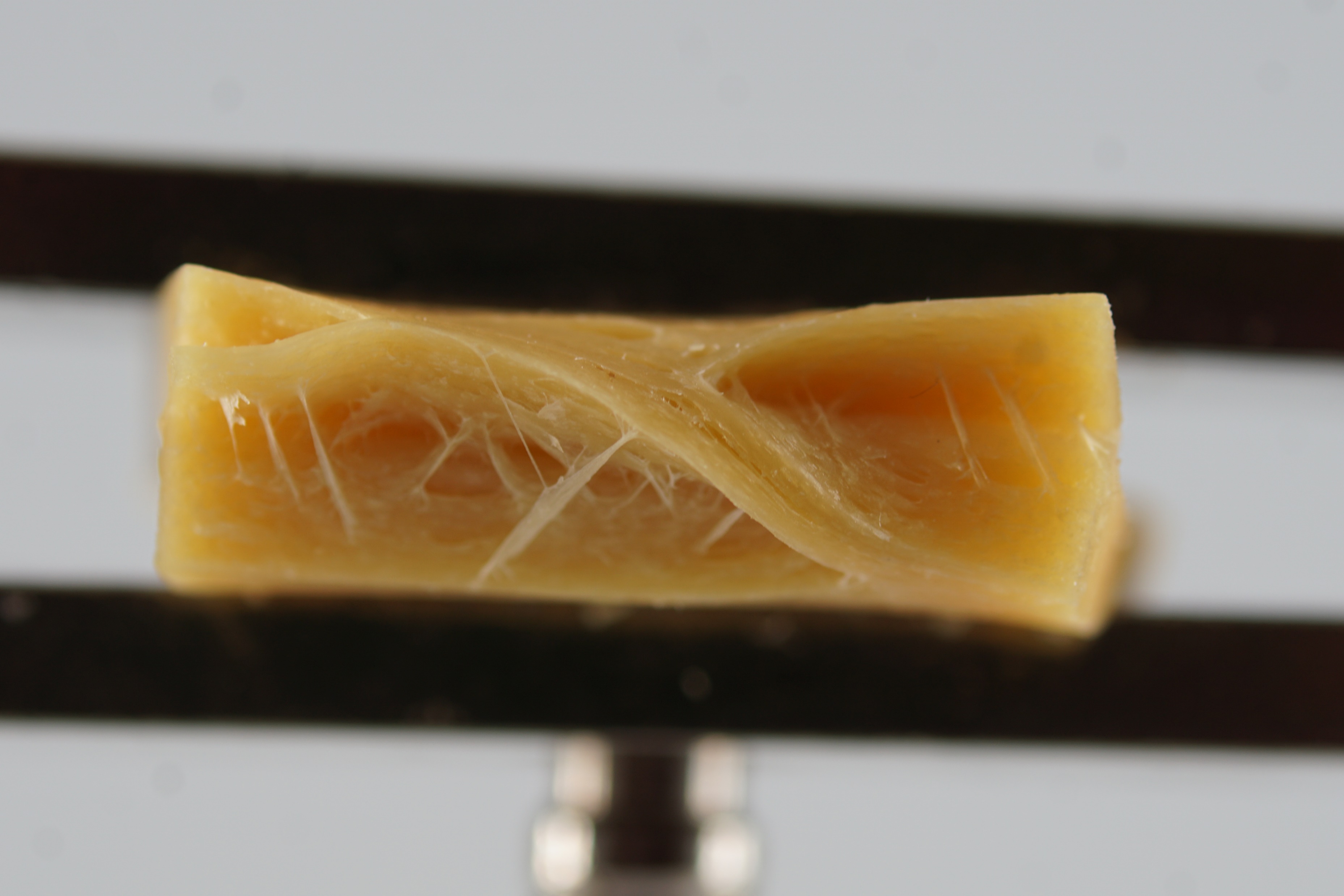

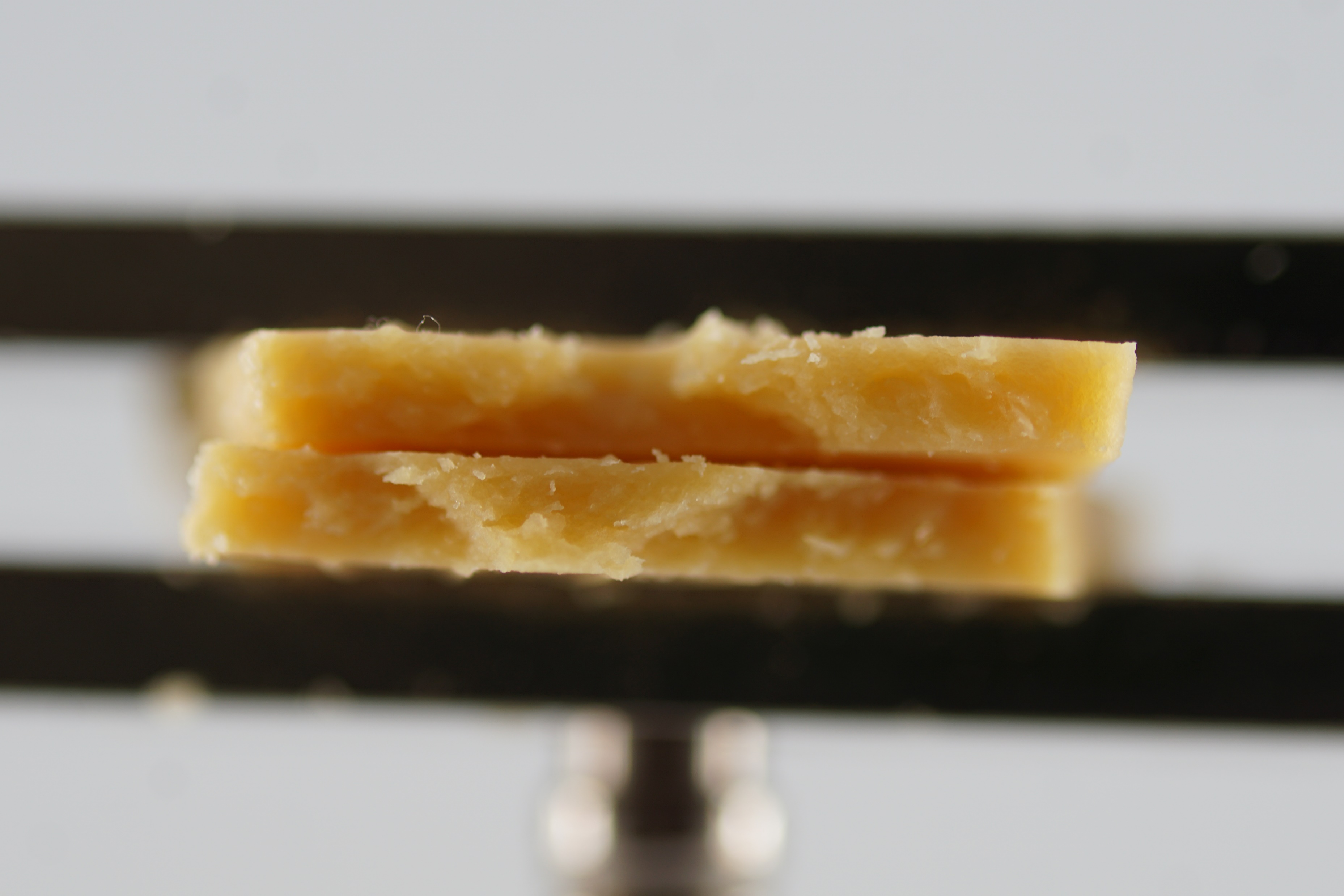


SPI-0-no shear

SPI-0

SPI-1

SPI-2.2

SPI-3.5

SPI-5

Figure S5.2: Macrostructural images of meat analogues folded open to expose the inner structure parallel to the shear direction. Samples are produced from soy protein isolate with different concentrations of pectin. Samples have been frozen after production and thawed before macrostructural analysis.


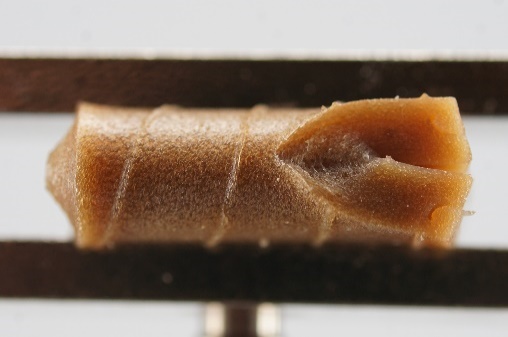

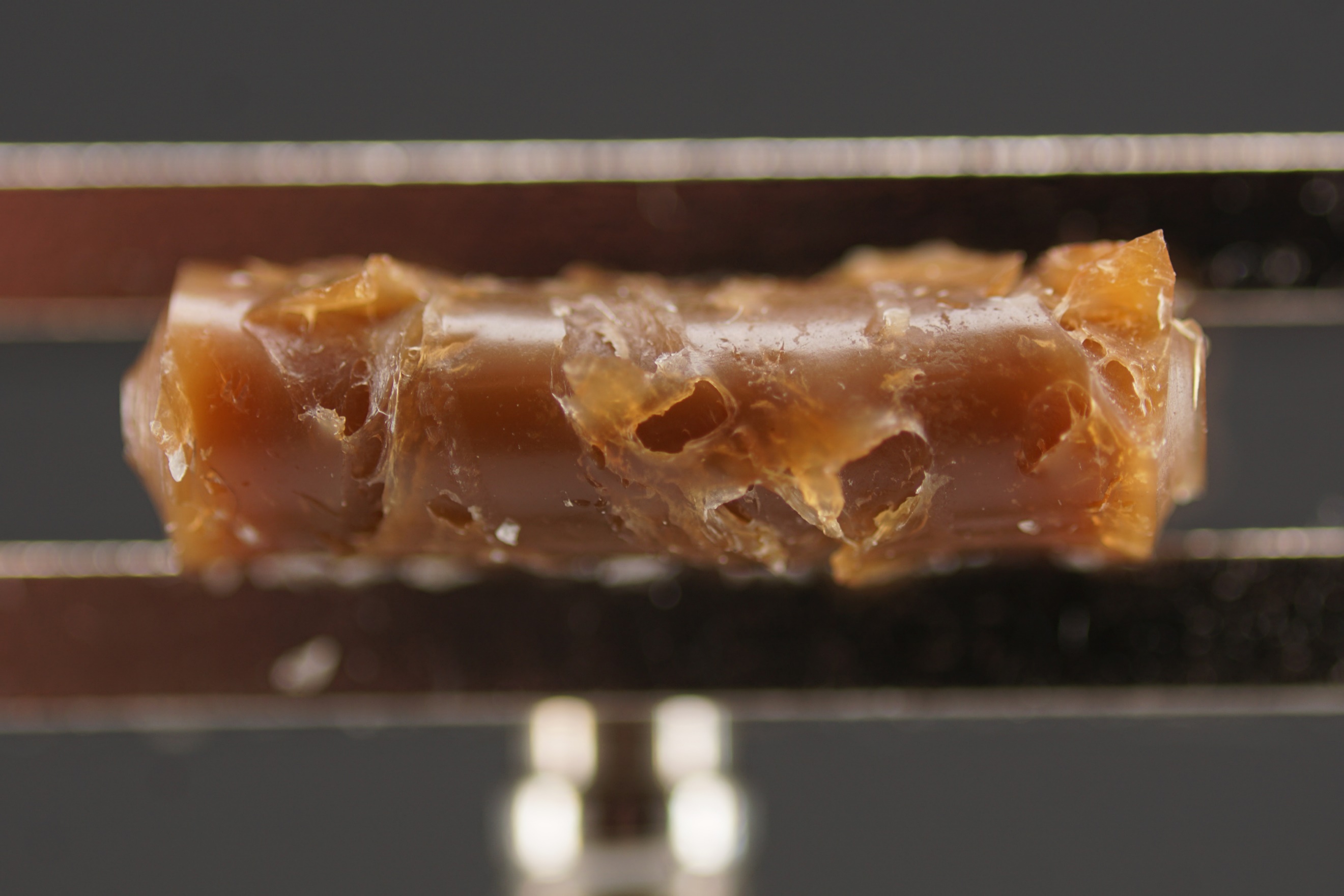

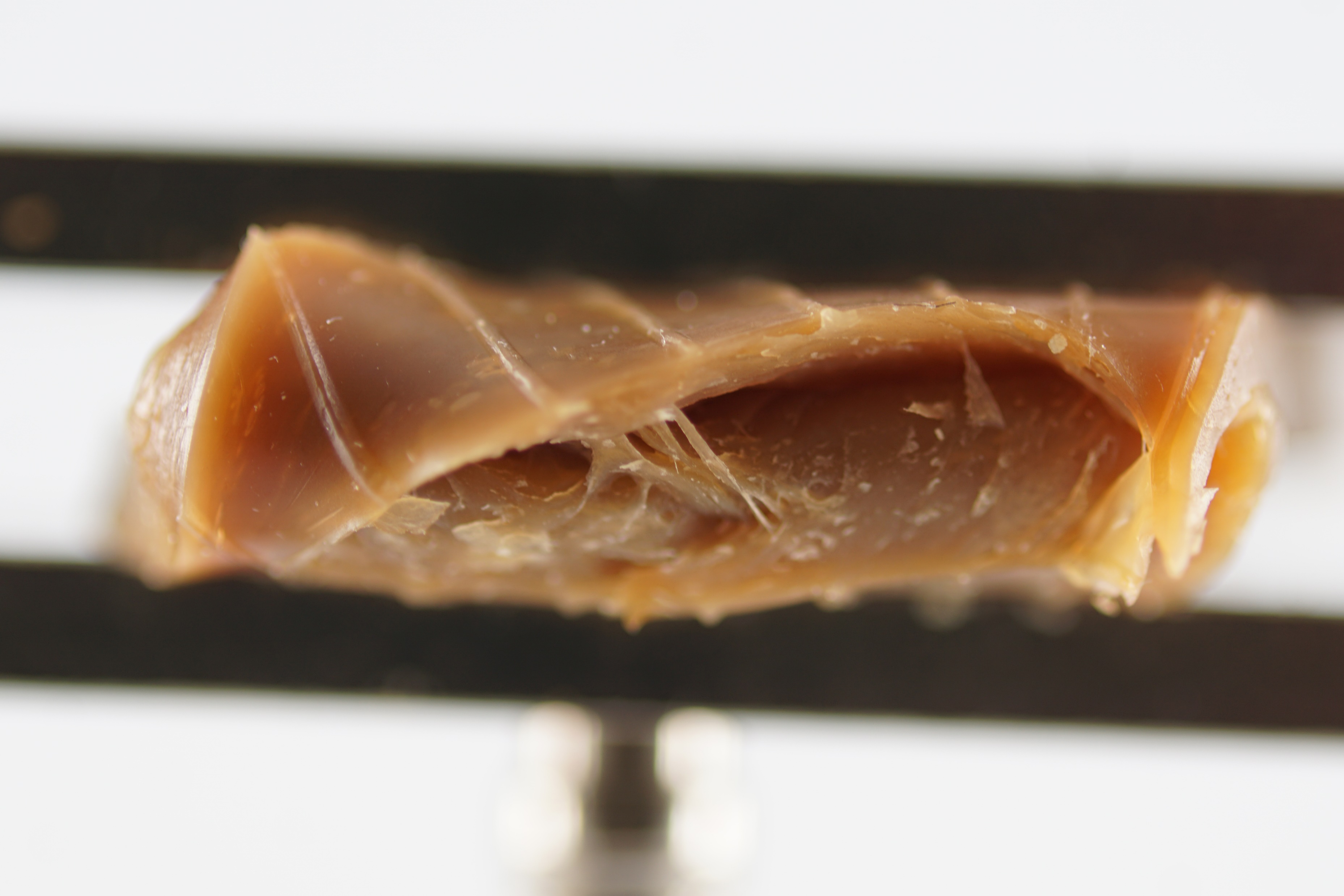

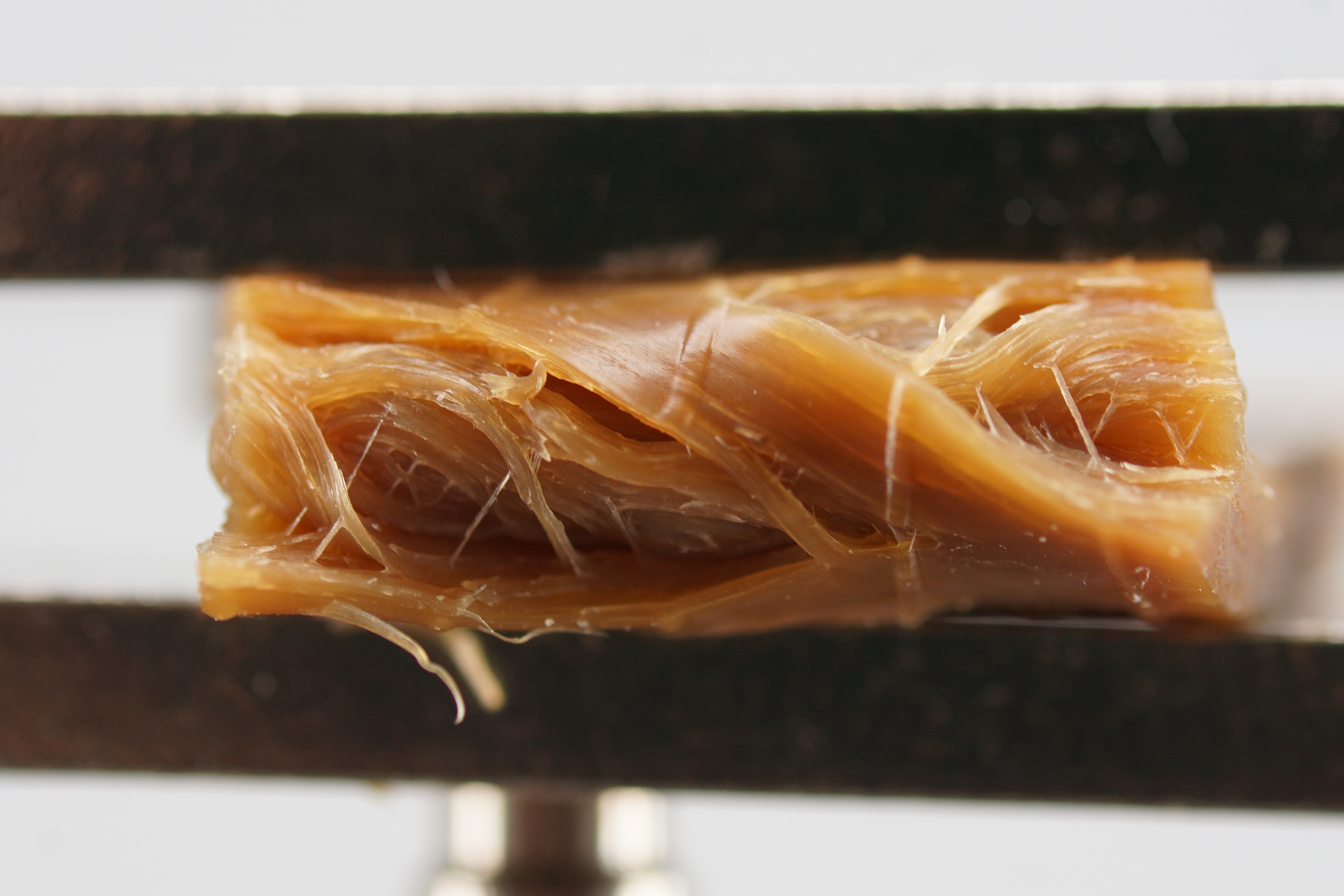

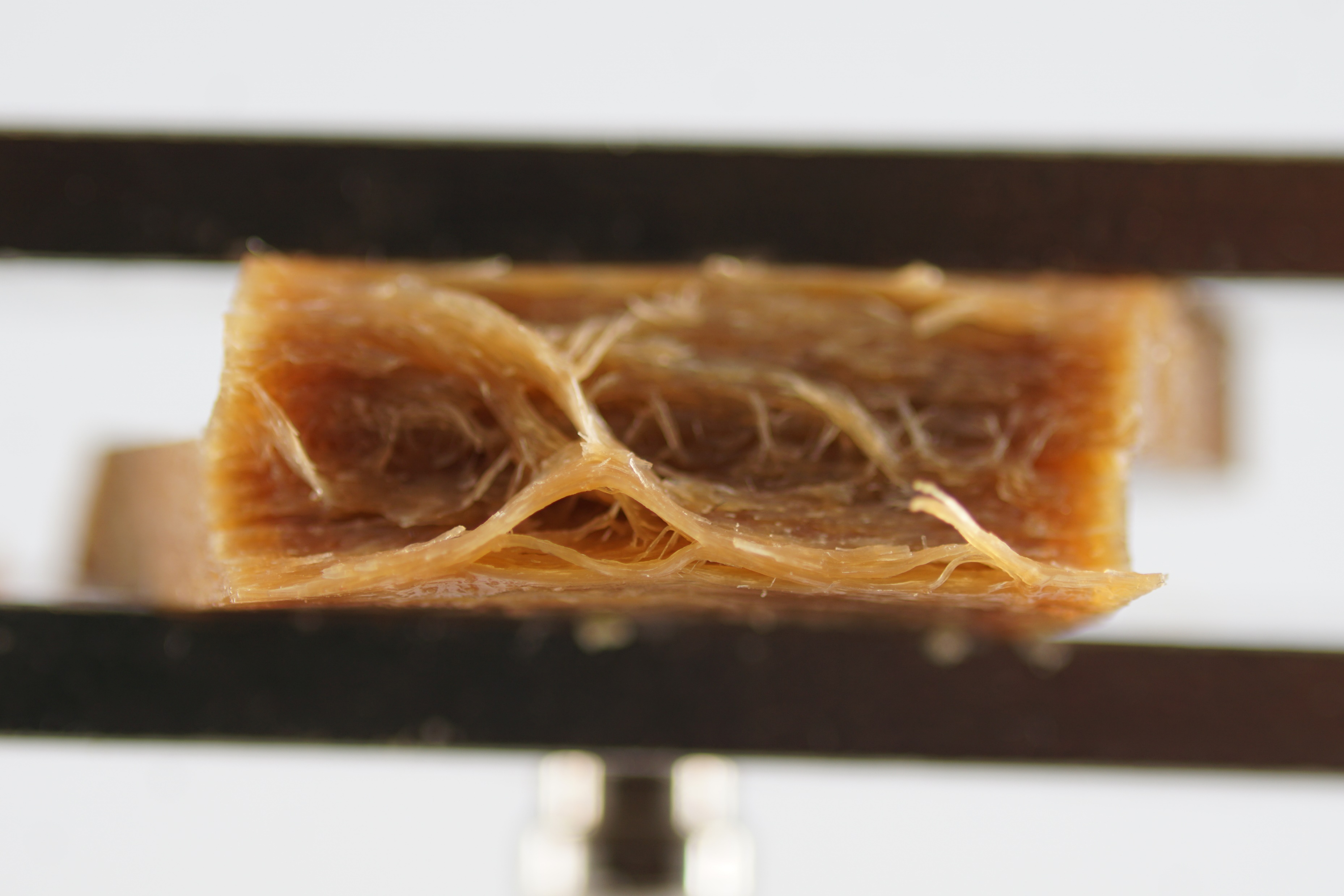

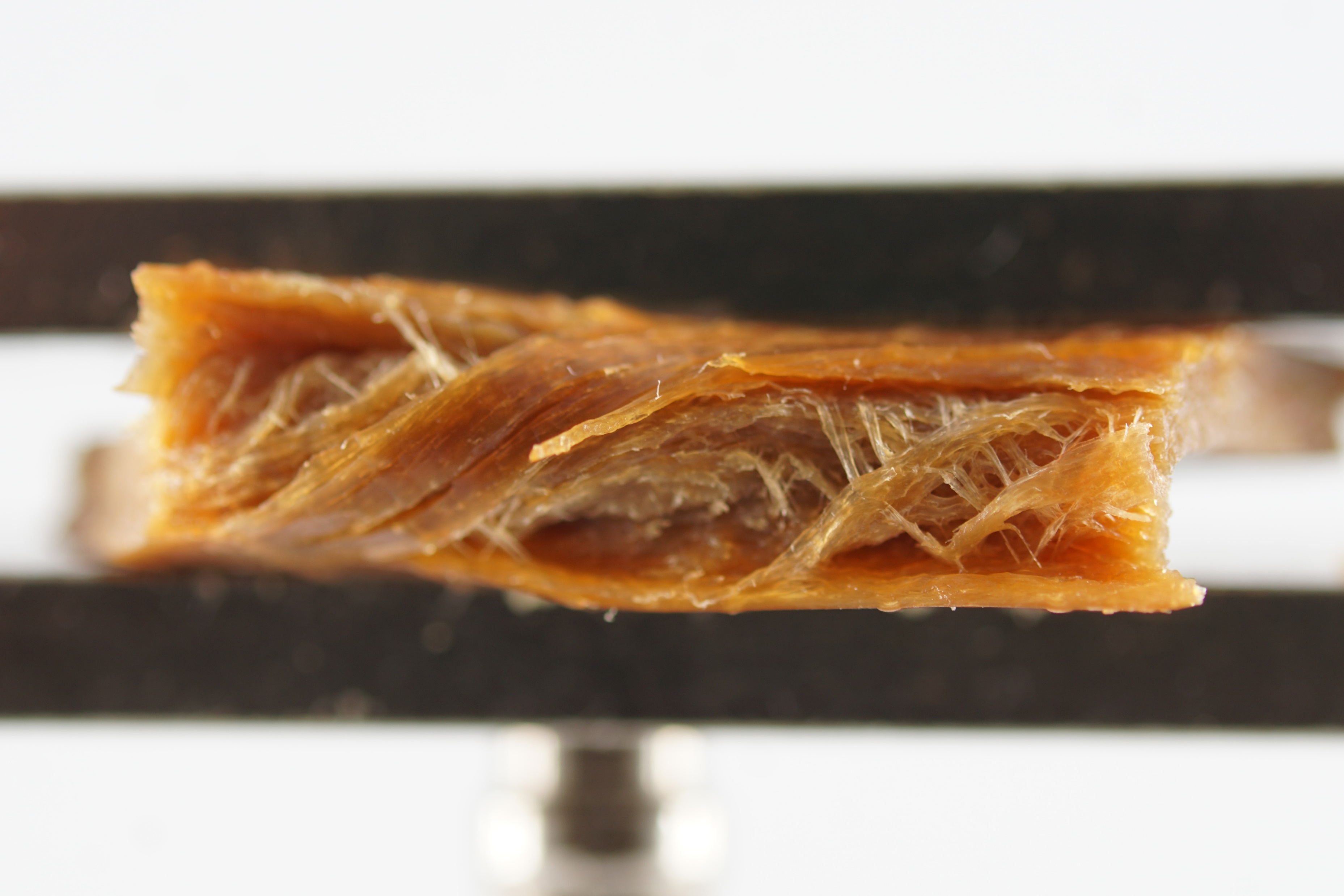


**Supplementary materials S6**

**A**

**B**

**C**

Figure S6.1: Stress-strain curves from single tensile specimen of A) MBFF 0 rpm, B) MBFF 15 rpm, and C) MBFF 30 rpm. Each stress-strain curve is calculated for a Poisson’s ratio of 0.5, 0.4, and 0.2.
